# A cross-regional examination of camelid herding practices in Peru from 900 BCE to 1450 CE: Insights from stable isotopes in camelid bone collagen and fiber

**DOI:** 10.1371/journal.pone.0306205

**Published:** 2024-10-14

**Authors:** Sarah J. Noe, Weston C. McCool, Kurt M. Wilson

**Affiliations:** 1 Department of Anthropology, University of California, Santa Barbara, Santa Barbara, California, United States of America; 2 Department of Anthropology, University of Utah, Salt Lake City, Utah, United States of America; 3 Society, Water, and Climate Research Group, University of Utah, Salt Lake City, Utah, United States of America; 4 Department of Geography, University of Utah, Salt Lake City, Utah, United States of America; New York State Museum, UNITED STATES

## Abstract

The economic, socio-political, and cultural significance of camelids in the Andean region is well-recognized, yet an understanding of their management evolution over pre-historical periods remains limited. This study aims to fill this gap by conducting the first cross-regional assessment of camelid pastoralism in Peru from 900 BCE to 1470 CE, using stable carbon and nitrogen isotopic compositions from the bone collagen and fibers of 577archaeological camelids across 21 sites. This research investigates the spatio-temporal shifts in camelid dietary habits, focusing on how the rise of intensive agriculture may have influenced change and led to the evolution of distinct roles for camelids in coastal versus non-coastal Andean economies. Our analysis indicates an increase in δ^13^C values over time on the coast, suggesting a shift towards maize-based camelid diets. Conversely, δ^13^C values decrease over time in highland environments, suggesting camelids consumed relatively more wild C3 forage and/or cultivated crops such as tubers. The study also reveals a significant positive relationship between latitude and δ^15^N values, suggesting increasing environmental aridity enriches δ^15^N in bone collagen. After controlling for this latitudinal effect, we observe a rise in δ^15^N values in both coastal and non-coastal camelids, suggesting that in later periods camelids may have been foddered in agricultural fields that were enriched with guano or dung fertilizer used to intensify production. Importantly, this research uncovers a distinct dietary divergence between coastal and inland camelids. The observed divergence in diets suggests contrasting socio-economic uses of camelids, where coastal camelids were predominantly involved in ceremonial and political activities, while those in non-coastal areas were crucial to the subsistence economy.

## Introduction

The camelids llama (*Lama glama*) and alpaca (*Vicugna pacos*) are the only livestock indigenous to the Americas and have played integral roles in both the economic and socio-political facets of ancient and contemporary Andean societies [1–5]. Their multifaceted roles in society included providing essential resources like meat, leather, and fiber; acting as pack animals in caravans; and supplying dung for fertilizer and fuel [4]. Today, camelid pastoralism is predominantly confined to the puna ecozone above 3,500 masl [6–9]. Pre-historically however, camelid herding in lower elevation areas was deeply integrated into the political economy for ceremonial uses, contrasting with its role in the highlands where it was a key element of the subsistence economy, acting as a risk mitigation strategy through diversification [10–12]. The advent of specialized pastoral practices and the development of intricate trade networks, rooted in the principle of Andean verticality, highlight camelids’ indispensable role in fostering the emergence of complex societies [1, 13, 14]. However, despite significant localized research [9, 10, 14–22], there remain few studies capturing cross-regional trends and variation indicative of divergent herding practices and camelid behaviors. Here we use stable isotope measures from 577 archaeological camelids from 21 sites to evaluate spatio-temporal changes in camelid dietary habits, mobility, and environmental interactions.

The analysis of stable isotope data can yield insights into wide-ranging themes such as human-environment adaptations, agricultural evolution, and socio-economic shifts [10, 15, 17, 21, 22]. In this context, the isotopic analysis of stable nitrogen and carbon is a valuable tool, with the potential to illuminate complex and dynamic human-camelid relationships. While the existing body of research has provided valuable insights into specific aspects of camelid herding, it has often focused on localized settings. There remains an opportunity to extend these findings through a cross-regional synthesis that examines variations in herding strategies across diverse temporal periods and ecological zones in Peru [14, 19]. Prior studies have formulated distinct regional dietary hypotheses—namely, that highland camelids predominantly consume C3 plants while coastal camelids are more reliant on C4 plants. These hypotheses, however, have typically been explored within relatively narrow geographical or temporal frameworks. Our study aims to bridge these gaps by comprehensively testing these hypotheses across multiple regions and time periods, thereby offering new perspectives on the practices and adaptations of Andean pastoralism from900 BCE and 1450 CE.

Building on foundational research that has explored diverse herding strategies across Peru, this study aims to trace the evolution of camelid herding practices from the Early Horizon through to the Late Intermediate Period, investigating how herders adapted to varying ecological and cultural pressures. This synthesis addresses critical questions about changes in camelid diet and management in response to the intensification of maize (*Zea mays*) agriculture and examines whether isotopic data support the differentiated roles of camelids in coastal versus highland settings. Specifically, we will address two central questions: 1) How did camelid diet and, by proxy, herding behavior change from 900 BCE to 1470 CE as intensive maize (*Zea mays*) agriculture became more prevalent? 2) Do stable isotope values from camelid bone and fiber support prior work suggesting camelids served different functions between coastal and non-coastal economies? We will also investigate a related methodological issue by evaluating whether we can detect nitrogen isotopic enrichment in camelid bone collagen as a function of environmental aridity proxied by latitude. Examining these questions reveals significant insights into the changing roles of camelids in agricultural practices and broader societal structures.

### Geographic background

**Fig 1:**
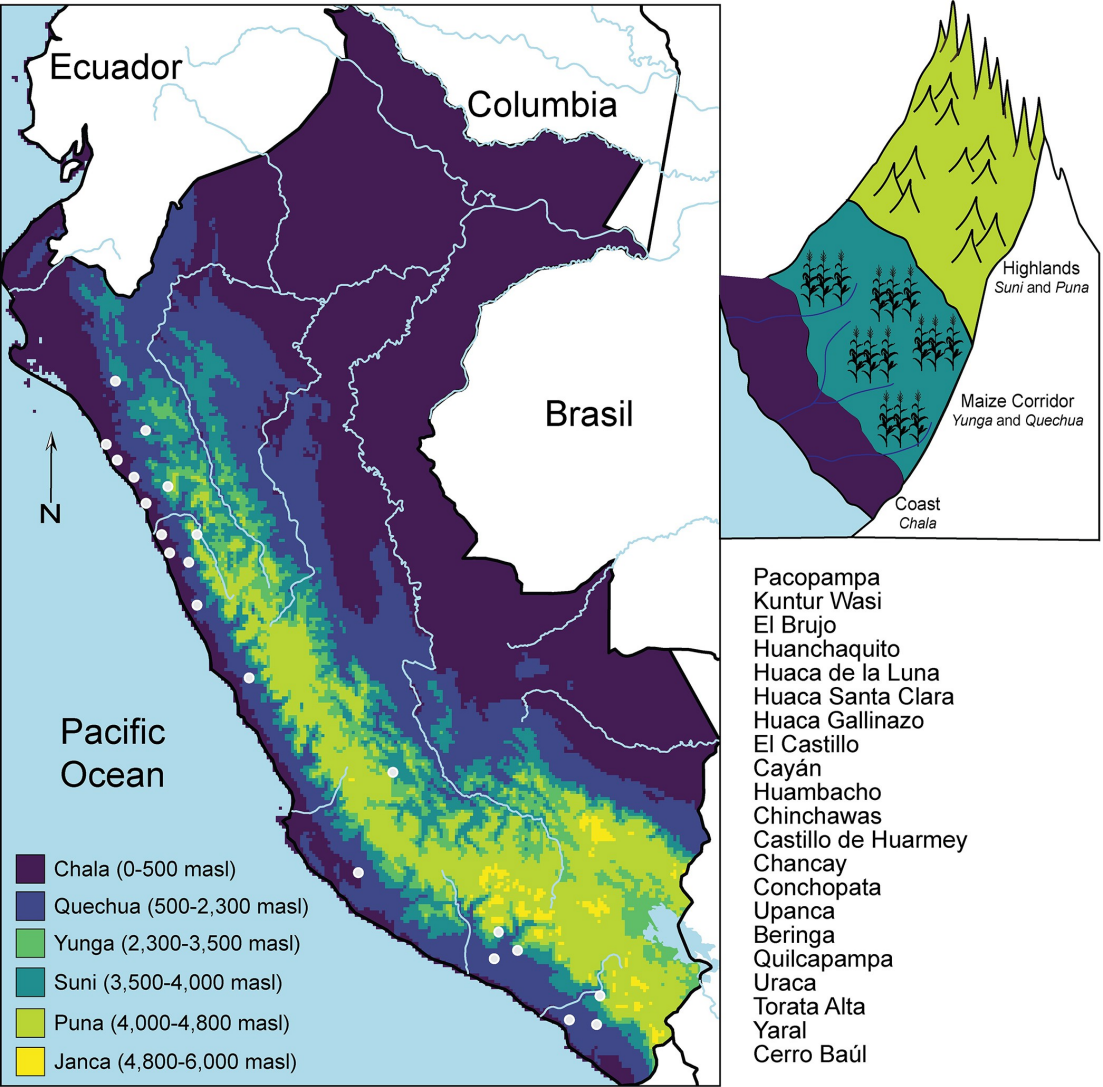
Ecological zones of Peru, ranging from the coastal lowlands (Chala) to the high-altitude mountains (Janca) [10]. The locations of key archaeological sites used in this study are mapped in a north-to-south progression, highlighting their specific altitudinal contexts [24].

Understanding past camelid herding practices requires characterizing the environmental conditions to which they are adapted. The Andean topography presents a distinct vertical succession of ecozones, with each elevation possessing unique characteristics and economic potentials (Fig 1). As highlighted by Sandweiss and Richardson III [23], these zones range from the arid Pacific coastline to the cold high-elevation sierras, with changes between zones characterized by decreasing daily temperatures and increasing annual precipitation as the elevation rises.

The *Chala* region on the western Pacific Coast, reaching up to 500 meters, is characterized by arid deserts interspersed with narrow, fertile valleys [25]. These valleys offer some grazing potential for camelids though the predominant arid conditions limit grazing sources, making it suboptimal for herding. Spanning elevations from 500 to 3,500 masl is the "Maize Cultivation Corridor" within the Andes, characterized by its more consistent rainfall and moderate climatic conditions conducive to intensive maize cultivation [1, 26, 27]. At the lower spectrum of this corridor, between 450 and 2,300 masl, is the Y*unga* ecoregion. This altitudinal zone is particularly suitable for the cultivation of maize (*Zea mays*), coca (*Erythroxylum coca*), and pink peppercorn (*Schinus molle*). While the *Yunga* does not serve as primary habitat for camelids due to the low density of grazing habitat as compared to the puna grasslands at higher elevations, its prolific agricultural yield offers potential fodder resources such as stored maize or maize stalks in post-harvest fields [2, 28]. The *Quechua* ecoregion, situated between 2,300 and 3,500 masl, offers a more temperate climate favorable for the growth of domestic staples, such as maize and potatoes. Historically, the *Quechua* zone has been a nexus of both agricultural and pastoral practices, supporting a significant camelid population that occasionally benefited from the region’s agricultural byproducts [29].

Higher in altitude is the *Suni* region, spanning 3,500 to 4,100 meters, which boasts rocky landscapes against the backdrop of a cold climate [30]. The harsher environmental conditions make large-scale agriculture challenging, favoring cold-resistant crops such as tubers. While camelids are able to navigate these rugged landscapes, the sparse vegetation demands more strategic herding practices. The *Puna* zone, situated above 4,100 meters, features expansive high-altitude grasslands and vast plateaus. This region is characterized by its cold temperatures and often harsh conditions, unsuitable for the majority of crops found in lower zones. However, the vast grasslands offer abundant natural fodder for camelids, making it their primary grazing habitat [2, 29, 31]. Beyond 4,800 meters lies the *Janca* region, the pinnacle of the Andes characterized by craggy mountain peaks and enduring cold and is unsuitable for human and camelid occupation.

With the rise of Andean pastoralism, camelids were herded across these varied ecozones, each offering a unique pasture and fodder profile [2, 13, 32]. Past work suggests that camelids consumed different resources depending upon where they were herded, including in some cases agricultural byproducts, with maize standing out as a prime example [9, 21, 22]. These varied feeding ecologies, reflective of differing herding practices, are captured in the isotopic signatures of camelids, enabling isotopes to provide insights into when and where camelid herding focused on wild vegetation versus cultivated fodder.

### Setting and archaeological context

Camelids, specifically llamas and alpacas, were the principal livestock of Prehispanic Andean society [4, 33]. Evidence from archaeological sites suggests that by around 4,500 BCE, early Andean herders had established corrals and seasonal rotational grazing patterns in the puna grasslands [4, 34]. As these practices matured, camelids were selectively bred not just for their meat, but also for their wool, which became a vital textile resource [18, 35]. By 3,000 BCE, the influence of, and practices associated with camelid herding had spread northward into the central Andes. This northward movement, enabled by the adaptable nature of camelids and their utility as beasts of burden, led to the development of expansive trade networks [1, 13].

By 2,000 BCE, camelids emerged as pivotal entities within Andean societies, not only serving economic and practical roles but also attaining symbolic and religious significance [36]. Several studies have provided localized descriptions of herding in the archaeological record [9, 10, 14–22]suggesting that altitude-defined ecological factors significantly influenced camelid management approaches [19, 31].

Isotopic analyses of both archaeological and modern camelids from high-altitude settings have been relatively scarce. From the limited studies available, it is generally proposed that above 3500 meters above sea level, specialized pastoralism involved the full-time management of camelid herds with minimal crop production[13, 37]. These highland herders are thought to have frequently traded with lower elevation communities for necessary crops [1, 11, 38]. Agropastoralism, which combines herding with the cultivation of frost-resistant tubers and grains, is proposed to have been a strategy to mitigate the challenges of the highland environment, characterized by limited arable land and frequent frost events.

Initial archaeological research on camelid herding was primarily focused on the highland pastoral systems, specifically the mid-altitude Quechua and Yunga highland zones, ranging from 500 to 3,500 masl. In these regions agropastoralism necessitates a trade-off between agricultural activities and pastoral responsibilities. The challenges in these areas include preventing overgrazing, necessitating frequent herd movements, and managing time allocation trade-offs between agriculture and herding [2, 32]. During times of agricultural scarcity, the reliance on crops to sustain herds may have compounded economic difficulties, potentially making maize-dependent herds a less viable long-term strategy [4, 11, 39].Localized isotopic studies conducted in Peru’s maize belt from the Early Intermediate Period to the Late Horizon corroborate this agropastoral model, showing that camelids primarily grazed on C3 plants, aligning with their minimal reliance on cultivated crops for fodder [14, 20–22].

In more recent years, the majority of research on camelid herding has been concentrated in coastal regions. Along the coast, from 0 to 500 masl, where there is a scarcity of forage, subsistence herding is thought to have played a more restricted role [10, 17]. Isotopic analyses show that camelids grazing on coastal or low-altitude wild vegetation have higher carbon isotopic values, likely due to a predominance of C4 plants in their diets and significantly higher nitrogen isotopic values resulting from the arid conditions of these areas [40]. The limited vegetation along the coast restricts camelid herding, leading to a reliance, primarily or exclusively, on agricultural products or byproducts for fodder. The exact scope and stability of maize as a primary component in camelid diets across ancient Andean societies remain subject to ongoing investigation and debate [9, 19, 34, 41, 42]. Contrary to prevalent belief, diets heavily reliant on maize may not have been economically viable within subsistence agropastoral economies, particularly in the maize-belt. Heavy dependence on maize makes these systems vulnerable to crop failures, which could lead to simultaneous declines in herd viability in these unpredictable environments if maize were principal fodder for camelids [2, 11, 39]. Additionally, while there is substantial evidence of camelids transitioning from basic dietary staples to playing multifaceted roles within the political economies of coastal areas, the full nature and extent of this transformation are still being extensively studied [16, 19, 41]. The importance of camelids within the Andean economic, social, and cultural structures is clear, but a detailed understanding of how their management evolved across different historical periods and ecological regions—especially contrasting the highland agropastoral systems with the coastal political economies—remains underdeveloped.

### Questions and predictions

Based on foundational isotopic studies of camelid herding strategies, we present two primary, and one secondary research questions regarding camelid isotopic compositions to analyze variations in herding practices across different ecological zones and specific historical periods.

Question 1: Is there a detectable dietary shift in camelids from 900 BCE to 1470 CE that correlates with the intensification of maize agriculture, and do these shifts manifest consistently between coastal and highland regions across different cultural periods?

Prediction 1: We anticipate that, correspondent with the growing importance of maize from the Early Horizon (900–200 BCE) through the LIP (1000–1470 CE), there will be a progressive shift in herding strategies towards the increased use of maize as fodder. This change is expected to result in progressively enriched δ^13^C values in camelid bone collagen and fur fibers across both coastal and highland ecozones.

Question 2: Does the rise of maize cultivation from the Early Horizon (900–200 BCE) through the LIP (1000–1470 CE) promote the practice of camelid foddering on enriched maize and other agricultural products? Prediction: We expect that increasing reliance on maize agriculture should incentivize increased use of dung and guano fertilizers, which would enrich the nitrogen content of maize and the nitrogen values of camelids foddered on enriched maize and other agricultural products.

Question 3 Do stable isotope analyses of camelid bone and fiber provide corroborative evidence of camelids fulfilling distinct roles in coastal versus non-coastal economies of ancient Andean societies?

Prediction: We hypothesize that the potentially varied role of camelids significantly changes the isotopic signatures between coastal and non-coastal economies in ancient Andean societies. On the coasts, where camelids played a relatively more symbolic and ceremonial role, we predict less variation in their dietary isotope signatures due to controlled feeding practices aligned with their status and use in rituals and state functions. We also expect coastal camelids to exhibit a more enriched maize isotopic signal as these camelids may have been foddered on domestic crops rather than wild C3 grasses. Conversely, in non-coastal economies, greater dietary variability is anticipated, as are relatively depleted carbon isotopic values indicative of consumption of wild C3 grasses, reflecting utilization of camelids as a subsistence economic diversification strategy for offsetting potential crop failure.

## Materials and methods

To evaluate our questions, we compiled a cross-regional dataset of camelid stable carbon and nitrogen isotope values from llama and alpaca, covering the period from 900 BCE to 1470 CE, derived from published sources across Peru (S1 File). No permits were required for the described study, which complied with all relevant regulations. From this novel dataset, we incorporated only direct isotope readings from bone collagen and fur fiber from camelids omitting isotopes obtained from textiles and from dentine. Isotope values from camelid bone and fiber were integrated into a single dataset following Szpak et al. [43] correction of +1.3 ‰ for the fiber samples. Any isotope value with a C:N ratio deviating from the range of 2.9 to 3.6 was excluded to maintain data quality [44]. Samples lacking reported carbon-to-nitrogen (C:N) ratios are included in the analysis, provided that the primary researcher confirmed in the original research that all documented samples fell within the acceptable C:N range. Further refinement led to the exclusion of samples associated with wild camelids, namely vicuña and guanaco, to focus research on pastoral contexts of domesticated camelids. To evaluate the potential for weaning effect influences on nitrogen, Kruskal-Wallis tests with subsequent pairwise tests were used to assess the differences in nitrogen values between adults and juveniles within each site, revealing that none of the sites showed a statistically significant difference in δ^15^N values between the two age categories, as all p-values exceeded the typical significance threshold of 0.05 (see S2 File). We therefore keep juvenile camelids in the analysis and the finalized dataset (n = 577) presents all of the reliable, directly comparable, carbon and nitrogen isotope values from domesticated llamas and alpacas.

### Stable isotope analysis

To elucidate the historical dietary patterns and herding practices of camelids in Peru, we conducted analyses focusing on δ^13^C and δ^15^N values from collagen extracted from bone and keratin from fiber [45]. The carbon (δ^13^C) and nitrogen (δ^15^N) isotope compositions in consumer tissues mirror the isotope profiles of their diet during tissue formation [44, 46–49]. The type of tissue influences stable isotope values. Keratin in fiber, such as hair or wool, forms during the maturation of each strand and does not undergo subsequent remodeling [18, 43, 50]. Given the rapid growth rate of these fibers, their isotopic signatures capture dietary and environmental conditions over shorter periods, perhaps spanning weeks to months. This contrasts with bone collagen, which offers a longer-term perspective. In bones, collagen undergoes remodeling in response to factors like tissue type, growth, and external stimuli [10, 19]. Prior studies indicate that although there might be minor variations in the diets of individual animals these fluctuations are unlikely to markedly alter long-term indicators, like the isotopic composition of bone collagen [48]. Therefore, seasonal changes in camelid diet may only be captured by assessing fiber isotope values [45] while bone provides the adult average diet.

Plant δ^13^C values are primarily determined by their photosynthetic pathway, resulting in different carbon isotopic compositions [44, 51]. The C3 pathway leads to discrimination against 13C, yielding lower δ^13^C values. In the Andes, the significant elevation gradient spans from tropical climates at lower elevations to cold alpine conditions at higher altitudes, leading to a broad range of δ^13^C values in plant isotopes due to varying environmental factors [52]. Temperature, humidity, atmospheric pressure, and differences in precipitation and soil types all influence isotopic fractionation; for instance, higher elevations generally have cooler, wetter conditions resulting in less fractionation and lower δ^13^C values, whereas lower, warmer, and drier areas see increased water stress, contributing to higher δ^13^C values [53]. These variations are further complicated by plant type differences; grasses and shrubs are typically more enriched in δ^13^C, while subshrubs present lower values, influenced by root distribution and leaf traits such as cuticles and stomatal density [54]. Lichens specifically can exhibit higher δ^13^C values as they lack stomata, reducing stomatal-related fractionation [55]. Additional local effects such as salinity near coastal areas can increase δ^13^C values, deviating from broader regional patterns. This combination of factors leads to C3 plants presenting with a 13C range of −35‰ to −20‰ in the region [55].

In contrast, the C4 pathway incorporates 13C more efficiently during photosynthesis, resulting in higher δ^13^C values in plant tissues. In the Andean region, C3 plants are prevalent; however, the landscape also hosts C4 species [56]. According to Szpak et al. [56], δ^13^C values show distinct variations: wild C3 plants average –26‰ within a range of −35‰ to −20‰, C4 plants average –12‰ ranging from −15 to −7‰. In the Andes, C3 plants, constituting the common highland grasses used as fodder, contrast with C4 plants, which, including maize alongside certain Andean amaranths (Amaranthus sp.), are more suited to warmer, arid conditions [57]. The carbon isotope composition of animal proteins, such as collagen found in bone and dentin, and keratin in hair and nails, largely mirrors the protein portion of their diet [45]. This is because essential amino acids are directly transferred from the consumed food to the body’s tissues. However, this relationship is not directly proportional. Typically, the δ^13^C values in the collagen of large herbivores are approximately 5‰ higher than the average values found in their diet [48, 58].

CAM plants, including cacti, succulents, and epiphytes, utilize Crassulacean Acid Metabolism, a photosynthetic pathway that results in intermediate δ13C values ranging between −22 and −10‰ [40]. As browsers, camelids may consume a mix of C3, CAM, and C4 plants, averaged δ^13^C values camelids consuming CAM or mixed C3 and C4 diets could potentially look similar isotopically [59]. However, most CAM plants in the Andes are succulents and cacti and were not a significant part of highland camelid diets [15, 60–63]. On the coast, local lomas vegetation can include a few CAM plants like *Tillandsia*, but the dominance of C3 plants in this system suggests limited consumption of wild CAM or C4 plants by coastal camelids. As such, δ^13^C values from camelid bone collagen primarily differentiate between C3 and C4 plant consumption. The prevalence of C3 and C4 plants in the Andes varies with altitude and rainfall; at higher altitudes (above 3500 meters above sea level), where rainfall can reach up to 400 mm per year, the vegetation is predominantly composed of C3 plants [40, 47, 64]. This is particularly evident in the puna ecosystem, characterized by bofedales—wetlands that provide both water and vegetation, creating ideal grazing areas for camelids [65]. C3 plants are also dominant in various ecological zones across northern Peru and in the central and south-central Andes. At lower altitudes, there is a shift in plant composition, with C4 species favoring areas of lower elevation and presumably warmer, drier climates [40]. In Peru’s lowlands, particularly the hyperarid coastal regions, suitable areas for camelid pasturing are scarce, mainly found near beaches, along river courses, and in lomas, or ’fog oases,’ located in the Andean foothills [15]. These lomas, flourishing seasonally due to ocean fog humidity, comprise various species of grass, shrubs, and trees. A wild C4 species, Distichlis spicata (Grama salada), is known to grow in coastal areas, forming extensive patches in certain locales. Valleys, fed by mountain water, provide natural grazing areas, including both coastal and highland (sierra) regions, though these same spaces tend to be the best locations for farming. Agricultural practices in these valleys involve managing irrigated fields for diverse crops like maize (C4), beans (C3), peanuts (C3), and avocados (C3). Camelids may have access to these crops, leading to the practice of foddering, especially with maize. This practice was previously suggested for the southern Peruvian highlands by Finucane et al. [9] and for the northern coast of Peru by Dufour et al. [10, 16].

In the Andean region, the altitude-related increase in temperature and precipitation notably affects the nitrogen isotopic composition in soils and plant life. Hot, arid environments, predominantly found at lower altitudes, are characterized by higher δ^15^N values [17, 43]. This is due to biogeochemical processes that cause the loss of the lighter nitrogen isotope, δ^15^N. These findings are supported by studies that connect these high δ^15^N values in plants, and subsequently in animal tissues, to the arid conditions and related nitrogen metabolic processes [66–68].

In contrast, at higher altitudes, plant tissues show comparatively lower carbon and nitrogen isotopic compositions. This variance is further complicated in agricultural plants, where fertilization plays a significant role in isotopic composition. Use of organic fertilizers, such as camelid dung, results in a moderate increase in δ^15^N values (+2‰ to +4‰), whereas seabird guano application can cause a substantial rise, often exceeding +20‰ [56, 69, 70]. As a result, animals grazing in coastal areas tend to exhibit higher nitrogen isotope values than those in higher altitudes, underscoring the complex relationship between fertilization methods, plant isotopic composition, and animal diets across different ecological zones [17]. In coastal Peru, for example, the irrigation of agricultural plants, such as maize, reduces the impact of water scarcity on δ^13^C and δ^15^N values [70]. Wild plants in these coastal areas have higher δ^15^N values, in contrast to irrigated agricultural plants, which generally exhibit lower δ^15^N values, even without fertilization [56]. Among terrestrial plants, only those that fix atmospheric nitrogen, such as legumes, exhibit distinctly unique δ15N values [40].

Due to prior suggestions of a latitudinal aridity effect on nitrogen levels, our research utilized generalized linear models (GLMs) to examine the relationship between Peru’s latitude (a proxy for increasing aridity along the north-south gradient) and camelid nitrogen isotope levels (δ^15^N). The initial Generalized Linear Model (GLM) analysis with latitude as a predictor showed a negative relationship between latitude and δ^15^N values, although the effect was not statistically significant (p = 0.109). Comparing this model to a null model using the Akaike Information Criterion (AIC) suggested only a marginal improvement. In a more comprehensive GLM incorporating additional environmental factors like elevation and ecozone, latitude still did not show a significant effect on δ^15^N values (p = 0.106), and the final model suggested no significant residual relationship between latitude and corrected δ15N values after accounting for these environmental influences (S2 File).

### Data preparation

Before implementing statistical analyses, we add several categorical variables to our data. In this study, we adopted Lanning’s [71, 72] standardized periodization of Andean prehistory to classify isotope values, assigning them to one of four cultural periods: Early Horizon (900–200 BCE), Early Intermediate Period (200 BCE-600 CE), Middle Horizon (600–1000 CE), and Late Intermediate Period (1000–1470 CE). This approach was utilized despite the existence of more specific cultural chronologies for certain regions, such as the Moche period in northern Peru, to maintain consistency with a widely recognized framework that facilitates broader comparisons across the Andes.

Secondly, we have divided camelid samples into two groups: coastal and highland. This categorization reflects a distinction in camelid herding based on ecoregion, as previously identified by researchers [14, 16, 19, 73]. Our study evaluates distinctions in camelid management by analyzing isotopic data across extensive regions and diverse cultural groups, enabling a detailed examination of these practices. This method allows us to explore the potential variations in herding strategies between and within coastal and highland societies, thereby addressing key questions about the influence of ecological context on pastoral practices.

### Analytical methods

To address our research questions, we adopted a mixed methods approach that integrates Kruskal-Wallis tests with subsequent pairwise tests, General Linear Models (GLM), and Linear Mixed Effects Models (LMER) [74]. We utilized the Kruskal-Wallis test to identify significant differences in δ^13^C and δ^15^N values across various periods and ecological zones, which is appropriate for our skewed data [74]. Detailed comparisons for specific periods were conducted using the Wilcoxon Rank Sum test. GLMs were used to analyze the impact of factors such as altitude and time on δ^13^C and δ^15^N isotopic compositions, while LMERs with random effects to control for latitude addressed spatial dependencies in data showing non-independent residuals [74, 75]. All statistical analyses were conducted in the R programming environment [74] (S2 File). These statistical tools allow us to delineate dietary transitions with precision, drawing on data from bone collagen and fiber to authenticate patterns and gauge the influences of geographic location and historical periods. Additional analyses include Kruskal-Wallis tests assessing δ^13^C and δ^15^N differences in the Bone and Fiber datasets and across cultural groups, a GLM examining the relationship between nitrogen isotopes and latitude, and visualizations that highlight isotopic variations driven by geographical and cultural changes over time (S2 File).

#### Research question 1: Carbon Isotopes, camelid diet, and maize agriculture

To explore question 1, initial Kruskal-Wallis tests assessed differences in *δ*^13^C values across periods and zones. Kruskal-Wallis tests are appropriate as the data is non-normally distributed data. Significant results led to detailed pairwise Wilcoxon tests to further explore specific differences between periods within each zone. We visualized these results using boxplots to clearly illustrate the data across periods and zones *[74]*. Additionally, we specified several Generalized Linear Models (GLMs) to investigate the impact of elevation (masl) and time periods on *δ*^13^C values, incorporating null, additive, and interaction models to evaluate the combined effects of these variables. Model fit was assessed using Akaike Information Criterion (AIC) and Bayesian Information Criterion (BIC), with visual regression plots enhancing the interpretability of interactions between altitude (masl) and period.

#### Research question 2: Nitrogen isotopes, camelid diet, and maize agriculture

Camelid nitrogen isotope (δ^15^N) values were categorized across ecozones and cultural periods, analogous to the division in the carbon analysis. An integrated dataset of bone and fiber samples was analyzed, considering variations across four cultural time periods. Initial Kruskal-Wallis tests were conducted to examine differences in corrected δ^15^N values across ecozones and periods as the data is non-normally distributed. Subsequent pairwise Wilcoxon tests were employed to explore specific differences between ecozones and periods. Furthermore, Linear Mixed Effects Models (LMERs) were developed to examine the relationship between elevation (MASL) and nitrogen isotopes [75]. A null model, including latitude as a random effect, was initially fitted to account for spatial variation. Subsequently, additive and interaction models were constructed to assess the individual and combined effects of MASL and period, with latitude as a random effect. Model adequacy was evaluated through Akaike Information Criterion (AIC) and Bayesian Information Criterion (BIC), with the results facilitating the selection of the most suitable model for interpreting the relationship between elevation and nitrogen isotopes.

#### Research question 3: Isotopic evaluation of varied camelid socioeconomic roles

To elucidate the varied socioeconomic roles of camelids, we synthesize the isotopic data obtained from Questions 1 and 2 within the existing body of scholarly research. This synthetic analysis interprets shifts in carbon and nitrogen isotope values in relation to camelid management practices, drawing on both our statistical findings and previously published evidence to explore the socioeconomic implications of camelid diets.

## Results

### Question 1: Carbon isotopes: Camelid diet and maize agriculture

The statistical analysis of our integrated dataset, using Kruskal-Wallis rank sum tests, highlights significant differences in δ^13^C corrected values across various ecozones and cultural time periods (Table 1). The analysis revealed pronounced disparities in δ^13^C values by ecozone, with a Kruskal-Wallis chi-squared of 152.25 (p < 0.0001). Focusing on regional comparisons, significant variances were noted across cultural periods within the coastal region. For camelids located along the coast, the Kruskal-Wallis test showed a chi-squared value of 101.85 (p < 0.0001), whereas for camelids from the highland region, the carbon isotope signature showed a chi-squared value of 27.316 (p < 0.0001).

**Table 1:** Summary of Kruskal-Wallis tests and pairwise comparisons for δ^13^C and δ^1^⁵N values.

**Kruskal-Wallis Tests**				
**Isotope**	**Location**	**Comparison**	**Chi-squared**	**p-value**
**δ^13^C**	Coast	~ period	101.85	< 0.001
**δ^13^C**	Highland	~ period	27.31	< 0.001
**δ^13^C**	Highland + Coast	~ ecozone	152.25	< 0.001
**δ^1^⁵N**	Coast	~ period	48.489	< 0.001
**δ^1^⁵N**	Highland	~ period	11.963	0.008
**δ^1^⁵N**	Highland + Coast	~ ecozone	22.14	< 0.001
**Pairwise Comparisons: Time Period**				
** **	** **	**Coast**	** **	** **
**Isotope**	**Period Comparison**	**EIP**	**LIP**	**Middle Horizon**
**δ^13^C**	**Early Horizon**	0.0185	< 0.001	< 0.001
**δ^13^C**	**EIP**	-	< 0.001	0.28
**δ^13^C**	**LIP**	-	-	< 0.001
** **	** **	**Highland**	** **	** **
**Isotope**	**Period Comparison**	**EIP**	**LIP**	**Middle Horizon**
**δ^13^C**	**Early Horizon**	< 0.001	< 0.001	0.0011
**δ^13^C**	**EIP**	-	0.052	< 0.001
**δ^13^C**	**LIP**	-	-	0.6362
** **	** **	**Coast**	** **	** **
**Isotope**	**Period Comparison**	**EIP**	**LIP**	**Middle Horizon**
**δ^1^⁵N**	**Early Horizon**	0.0028	< 0.001	< 0.001
**δ^1^⁵N**	**EIP**	-	< 0.001	0.71
**δ^1^⁵N**	**LIP**	-	-	< 0.001
** **	** **	**Highland**	** **	** **
**Isotope**	**Period Comparison**	**EIP**	**LIP**	**Middle Horizon**
**δ^1^⁵N**	**Early Horizon**	0.135	1.000	1.00
**δ^1^⁵N**	**EIP**	-	0.022	0.57
**δ^1^⁵N**	**LIP**	-	-	1.00
**Pairwise Comparisons: Ecozone**				
	**Isotope**	**Comparison**	**p-value**	
	δ^13^C	Highland—Coast	< 0.001	
	δ^1^⁵N	Highland—Coast	< 0.001	

**Fig 2:**
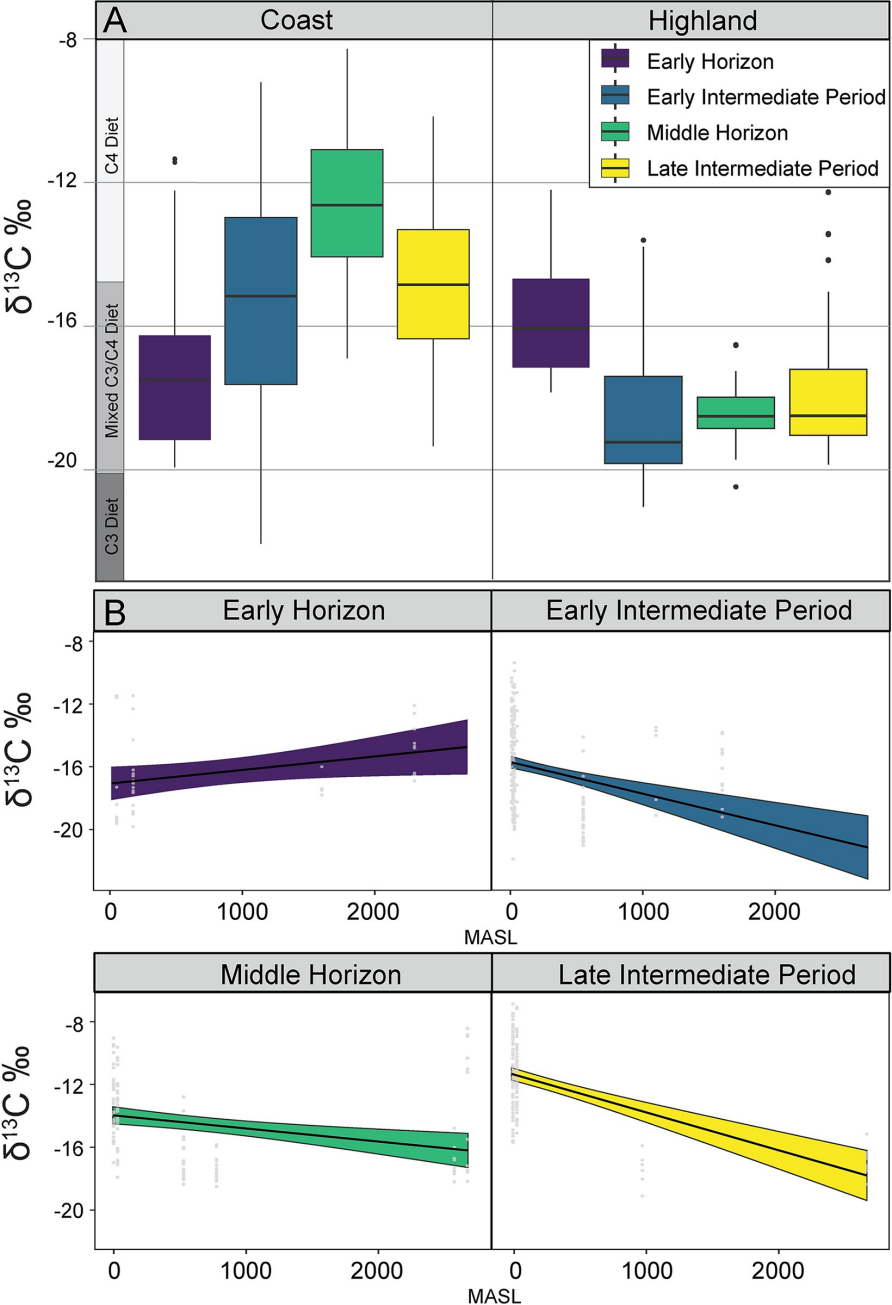
Carbon isotopes. Panel A shows boxplots of δ^13^C values in camelids from coastal and highland regions, organized by cultural periods: Early Horizon, Early Intermediate Period, Middle Horizon, and Late Intermediate Period. Panel B illustrates regression plots of δ^13^C values against elevation (MASL) for each cultural period, detailing the changes in isotopic composition with increasing altitude. Each regression is color-coded by period, with the shaded areas representing 95% confidence intervals, indicating the robustness of the linear model fit across the range of elevations.

Further distinctions were identified through Pairwise Wilcoxon rank sum tests with continuity correction, which provided detailed contrasts between specific periods. For camelids from coastal sites, significant differences were observed between all four cultural time periods (Table 1; Fig 2). In the highland region, comparisons of δ^13^C values showed significant differences between the Early Horizon and the three later time periods, but no significant variation was found between the EIP, Middle Horizon, and LIP (Table 1; Fig 2). Additionally, a substantial isotopic difference was observed between the highland and coast regions for δ^13^C values, underscoring the ecological influence on isotopic signatures (p < 0.0001). These results were confirmed to be robust as the Kruskal-Wallis and Wilcoxon tests held up under 1000 bootstrap iterations, indicating the results are not due to sampling bias. Detailed analyses and bootstrapping procedures are provided in the Supplemental Material (S2 File).

A Generalized Linear Model (GLM) was utilized to analyze the variation in δ^13^C values the integrated bone and fiber isotope dataset (Table 2). Initially, a null model was specified, demonstrating a significant mean level of δ^13^C values (Intercept = -15.2582, p < 0.0001). This indicates that the average δ^13^C value across the dataset is significantly different from zero. Subsequently, an additive model incorporating elevation (MASL) as a predictor revealed a significant negative relationship between elevation and δ^13^C values (Coefficient = -0.001157, p < 0.0001). This suggests that as elevation increases, δ^13^C values decrease, highlighting the influence of altitude on carbon isotope composition. The analysis was extended with a more complex interaction model that included the effects of elevation and specific time periods (early horizon, early intermediate period, middle horizon, late intermediate period). This model provided the best fit (AIC = 2794.4, BIC = 2833.85). The interaction model revealed significant interactions between elevation and time periods, particularly noting pronounced changes in δ^13^C values with elevation during the Late Intermediate Period (Coefficient for MASL: Late Intermediate Period = -0.003267, p < 0.0001). This indicates that the effect of elevation on δ^13^C values was more pronounced during this period. The GLM analysis reveals that increasing elevation is associated with decreasing δ^13^C values, and that this relationship is influenced by the time period. Specifically, during the Late Intermediate Period, the impact of elevation on δ^13^C values was more substantial compared to other periods.

**Table 2:** Comparative analysis of δ^13^C and δ^1^⁵N isotopes in camelid bone and fiber using Generalized Linear and Linear Mixed Effects Models.

**δ^13^C**						
**Test Type**	**Model Description**	**Coefficient Type**	**Value**	**Std. Error**	**t Value**	**p-Value**
GLM	Null Model	Intercept	-15.2582	0.1265	-120.6	< 0.001
	Additive Model	Intercept	-14.83	0.1385	-107.09	< 0.001
		MASL	-0.00116	0.000177	-6.528	< 0.001
	Interaction Model	Intercept	-17.07	0.5431	-31.426	< 0.001
		MASL	0.000862	0.000425	2.029	0.043
		MASL: EIP	1.37	0.5754	2.38	< 0.001
		MASL: Middle Horizon	1.078	0.6122	1.761	0.0788
		MASL: LIP	4.444	0.5797	7.667	< 0.001
**δ^1^⁵N**						
**Test Type**	**Model Description**	**Coefficient Type**	**Value**	**Std. Error**	**t Value**	**p-Value**
LMER	Null Model	Intercept	7.5845	0.2581	29.38	< 0.001
	Additive Model	Intercept	7.7816	0.3409	22.826	< 0.001
		MASL	-0.00027	0.000275	-0.979	0.328
	Interaction Model	Intercept	7.006	0.6824	10.266	< 0.001
		MASL	0.00015	0.000565	0.265	0.791
		MASL: EIP	1.2766	0.7899	1.616	0.107
		MASL: Middle Horizon	0.7772	0.7948	0.978	0.328
		MASL: LIP	1.3778	1.0796	1.276	0.202

### Question 2: Nitrogen isotopes—Camelid diet and maize agriculture

**Fig 3:**
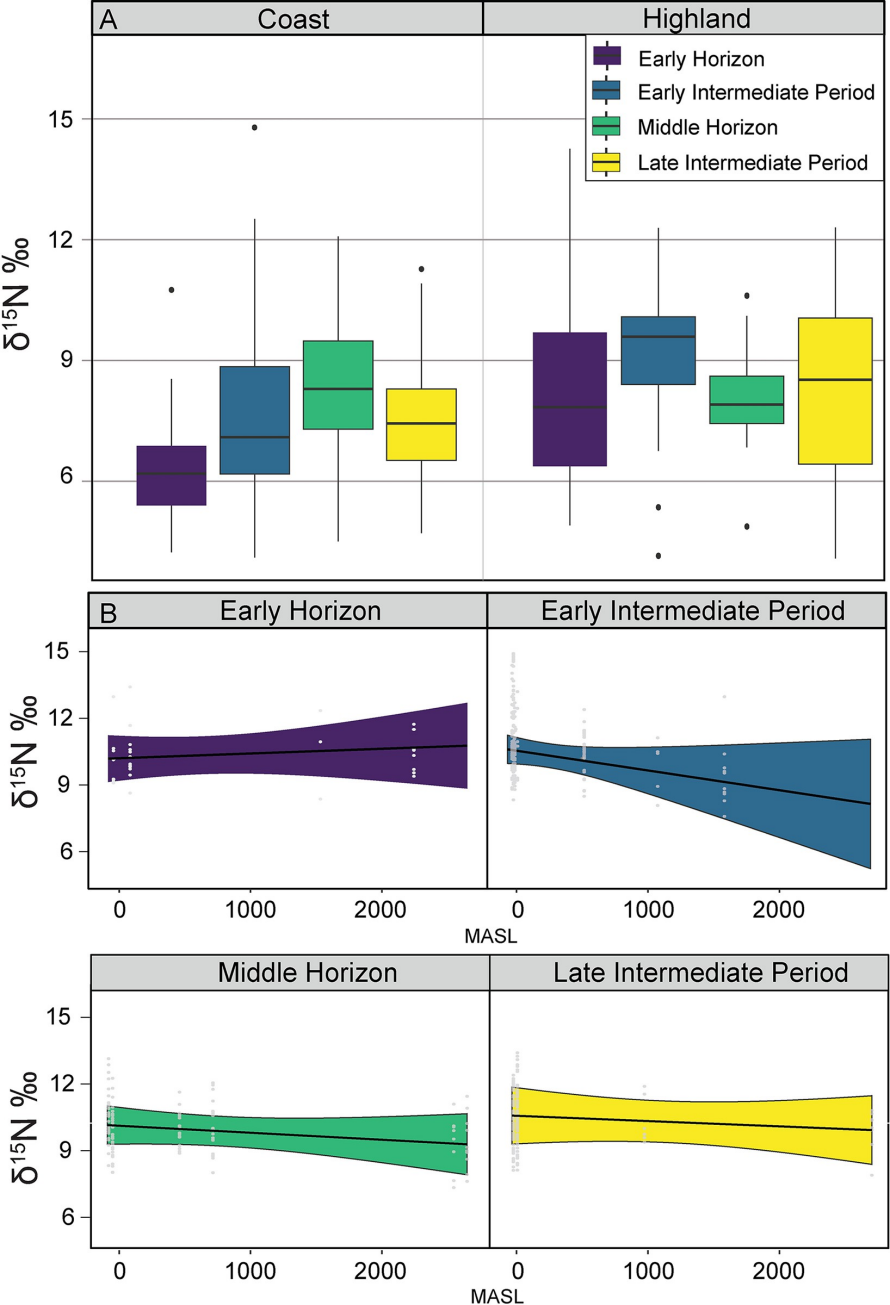
Nitrogen isotopes. Panel A shows boxplots of δ^1^⁵N values in camelids from coastal and highland regions, categorized by cultural periods: Early Horizon, Early Intermediate Period, Middle Horizon, and Late Intermediate Period. Pannel B illustrates regression analyses of δ^1^⁵N values against elevation (MASL) for each cultural period. The color-coded regressions, accompanied by 95% confidence intervals shown as shaded areas, underscore the relationship’s variability and the model’s predictive confidence at different elevations.

The investigation of δ^1^⁵N values, employing Kruskal-Wallis rank sum tests, revealed significant differences across both coastal and highland regions, as well as across various cultural periods (Table 1; Fig 3). For camelids from the coastal region, δ^1^⁵N levels varied significantly over time, evidenced by a Kruskal-Wallis chi-squared value of 48.489 and a significant p-value (p < 0.0001). In the highland region, δ^1^⁵N levels also showed significant variation by period (chi-squared = 11.964, p = 0.0075). Subsequent analysis using Pairwise Wilcoxon rank sum tests indicated pronounced differences in δ^1^⁵N values between the coastal camelids from each of the four periods. Specifically, significant differences were found between the Early Horizon and the Early Intermediate Period (p = 0.0028), the Late Intermediate Period (p < 0.0001), and the Middle Horizon (p = 0.0028). Additionally, significant differences were observed between the Early Intermediate Period and the Late Intermediate Period (p < 0.0001), and between the Middle Horizon and the Late Intermediate Period (p < 0.0001). In the highland region, pairwise comparisons revealed no significant differences between the Early Horizon and the Early Intermediate Period (p = 0.135), the Late Intermediate Period (p = 1.000), or the Middle Horizon (p = 1.000). A significant difference was found between the Early Intermediate Period and the Late Intermediate Period (p = 0.022), and a nearly significant difference between the Early Intermediate Period and the Middle Horizon (p = 0.057). No significant variation was found between the Late Intermediate Period and the Middle Horizon (p = 1.000). Comparisons between ecozones revealed a substantial isotopic difference between the highland and coastal regions for δ^1^⁵N values (p < 0.0001), underscoring the ecological influence on isotopic signatures. These results were confirmed to be robust as the Kruskal-Wallis and Wilcoxon tests held up under 1000 bootstrap iterations, demonstrating 99.9% success for the coast and 99.1% for the highland, indicating that the results are not due to sampling bias. Detailed analyses and bootstrapping procedures are provided in the Supplemental Material (S2 File).

Using Linear Mixed Effects Models (LMER), we examined δ^1^⁵N isotope variations across bone and fiber samples from various latitudes (Table 2; Fig 3). The null model showed significant average δ^1^⁵N values (Intercept = 7.5845, p < 0.0001), indicating that the mean δ^1^⁵N value across the dataset is significantly different from zero. An additive model including elevation (MASL) suggested that elevation alone does not significantly affect δ^1^⁵N levels (Coefficient = -0.0002689, p = 0.328). This implies that changes in elevation do not have a straightforward impact on nitrogen isotope values. Expanding to an interaction model revealed how elevation interacts with periods to influence δ^1^⁵N values. The interaction model showed that the effect of elevation on δ^1^⁵N values varies across different time periods. Specifically, the interaction term for elevation and the early intermediate period (MASL: EIP) was significant (Coefficient = -0.0017763, p = 0.012), indicating a more pronounced effect of elevation on δ^1^⁵N values during this period. The model also adjusted the baseline δ^1^⁵N values for different periods, with the intercept set at 7.006 (p < 0.0001). However, the interactions for the middle horizon (Coefficient = 0.7772, p = 0.328) and late intermediate period (Coefficient = 1.3778, p = 0.202) were not statistically significant. This analysis reveals that while elevation alone does not significantly influence δ^1^⁵N values, its impact varies across different historical periods. During the early intermediate period, higher elevations are associated with a notable decrease in δ^1^⁵N values. The interaction model’s complexity provided a better fit (AIC = 2383.021, BIC = 2426.822), illustrating the detailed interplay between environmental and temporal factors in shaping isotopic profiles.

## Discussion

This study systematically investigated three key questions concerning ancient camelid herding practices in Peru from 900 BCE to 1470 CE, focusing on the impact of the rise of maize agriculture on camelid dietary patterns. Building on the seminal works of Dufour [10, 16] and Szpak [12, 19], which documented flexible dietary strategies and regional differences in camelid diets, our analysis further elucidates the development of camelid herding practices through time in response to maize agricultural intensification. Our results reveal a pronounced divergence in camelid dietary patterns that not only underscores the influence of maize agriculture but also reflects distinct socio-economic differentiations between the coastal and highland regions. In coastal areas, camelids were integrated into ceremonial and political roles, consuming food via fodder that included a higher proportion of maize, aligning with findings from Szpak and Dufour’s research [16, 19]. Conversely, highland camelids primarily maintained a subsistence role in the consumption of C3 plants, as noted by Tomczyk [21] and Vásquez [76], showcasing regional adaptations to ecological and socio-political pressures. Here we discuss new insights into the development of socio-economic differences in camelid herding through time, driven by the intensification of maize agriculture.

### Question 1: Carbon isotopes: Camelid diet and maize agriculture

The analysis of δ^13^C values across various ecozones and cultural periods using Kruskal-Wallis rank sum tests and GLM provided substantial evidence for significant dietary shifts in camelid populations by region. These findings partially support Prediction 1, which posited that changes in isotopic composition would occur concurrently with the adoption of maize agriculture as a major economic activity and should vary by ecoregion. Regarding ecoregion, the δ^13^C values on the coast were significantly higher compared to those from the highland region, supporting the hypothesis of trans-regional differences in the adoption of maize-based foddering practices for camelids based on being coastal or non-coastal. Coastal camelids appear to have been foddered with maize to a significantly greater extent than non-coastal camelids overall.

Providing support for Prediction 1, significant temporal shifts in carbon isotopic values were observed in camelids from coastal sites, with substantial increases in δ^13^C values from the Early Horizon through to the Late Intermediate Period (LIP). These findings strongly suggest that coastal regions experienced a pronounced intensification of maize use as fodder over time, supporting the hypothesized link between the adoption of maize agriculture and the increase in carbon isotopic signatures in camelid diets [12, 19]. Coastal herders likely allowed the animals to graze on maize stalks in the fields, forage in cultivated plots, or brought maize stalks to the herds in corrals and pens [73, 77]. While the coastal increase in observed δ^13^C values appears to correspond with maize intensification temporally, there are plausible, if less likely, alternatives. The possible consumption of local loma vegetation, which was largely comprised of C4 and CAM plants could influence these isotopic profiles [40]. However, C3 plants dominate Peru’s flora, lomas vegetation is typically a seasonal and not year round phenomena, and it would likely be challenging for coastal herders to find sufficient wild C4 pastures to account for the enriched carbon isotope values observed in camelid diets.

There is also variation in coastal camelid diet, particularly in the Early Intermediate Period, and a decline in carbon values during the Late Intermediate Period. Although more individuals had diets with a high proportion of C4 plants, this does not fully capture the complexity and variability in the data. This variability and decline seem to reflect a long-term, mixed C3-C4 diet signal, suggesting a diachronic switch in diet, perhaps associated with changes in geographic location. Especially in the Late Intermediate Period, camelids may have been part of caravans moving to and from the coast, consuming a more mixed C3/C4 diet than those living exclusively on high-altitude pastures, with maize fodder provided at various points along the way [16, 73].

In the highland regions, detectable differences in isotopic values were only observed between the Early Horizon and subsequent periods, suggesting maize foddering in the Early Horizon but far less in later periods. Indeed, the pattern observed from the Early Intermediate Period through to the Late Intermediate Period shows that the camelid diet was predominantly C3-based, with the possibility of some C4 plant consumption. It is important to note that the small, localized sample size from the Early Horizon limits our comprehensive understanding of herding practices during this period. Therefore, it seems more parsimonious to suggest that highland camelids broadly maintained a consistent diet of primarily C3 plants over time, unaffected by the intensification of maize agriculture. While camelid groups from coastal sites generally exhibit greater isotopic variability compared to those from the highlands, there are some outlier camelids from each of the three later time periods. These outliers indicate a rare consumption of a C4 plant, such as maize, possibly reflective of movement between the coast and the highlands.

These results collectively demonstrate that regional and temporal variations in maize agriculture significantly influenced camelid diets, with ecological variation resulting from elevation differences playing a crucial role. The progressive enrichment of δ^13^C values in coastal regions suggests a stronger integration of maize into diets over time, contrasting with the highlands where camelids predominantly consumed non-maize plants [12, 19, 73, 77]. These adaptations to maize agriculture, although linked, occurred in non-uniform ways across different regions. Along the coast, where limited forage was available and camelids may not have been primarily used for subsistence, maize agriculture likely led to increased maize foddering [73, 77]. Conversely, despite the ecological suitability of C4 plants in the maize belt, camelids there exhibited lower δ^13^C values, indicative of a diet largely composed of C3 plants, which are abundant across all ecological zones. In highland areas, the increased adoption of maize seems to have led to a reduction in maize foddering, potentially to preserve fields for crop cultivation or as a risk mitigation strategy against crop failure [11, 39]. These findings underscore the responsiveness of past herding practices to the changing agricultural landscape, revealing that herders actively adapted their livestock’s diets in accordance with regional economic and agricultural conditions.

### Question 2: Nitrogen isotopes—Camelid diet and maize agriculture

Our study’s analysis of δ^15^N isotope values in camelid bone and fiber aimed to determine if nitrogen values changed over time, potentially as a result of changes in manuring and fertilizing practices associated with agricultural intensification. Controlling for the latitudinal aridity effect on nitrogen, our Linear Mixed Effects Models demonstrated that both elevation and time period play crucial roles in modulating δ^15^N levels. Camelids herded along the coast are typically thought to exhibit high δ^1^⁵N values because plant tissue δ^1^⁵N values tend to be higher under conditions of limited water availability [49, 78]. In contrast, highland plants receive more precipitation, and consequently, camelids feeding in the highlands are typically expected to exhibit lower δ^1^⁵N values than those at lower elevations. However, our data shows an intriguing opposite pattern: the average δ^1^⁵N values for highland camelids are generally higher than those for coastal camelids over time. This is an unanticipated result. It may suggest that highland camelids may have consumed more fertilized plants, which are enriched in δ^1^⁵N, while coastal camelids primarily grazed on unfertilized plants, resulting in lower δ^1^⁵N values.

For camelids from coastal regions, Kruskal-Wallis rank sum tests demonstrated marked variability in δ^15^N levels over time, culminating in significant differences, particularly between the Early Horizon and the Middle Horizon, followed by a slight decline in nitrogen levels during the Late Intermediate Period (LIP). This overall pattern suggests a pronounced increase in nitrogen values parallels the intensification of agricultural practices and increased consumption of maize by camelids, suggesting substantial maize foddering in coastal irrigated and/or fertilized fields [56, 79]. Indeed, the temporal correlation between the high δ^13^C and δ^15^N values indicates a diet of maize grown in fields fertilized with guano or manure which has been previously identified to increase δ^15^N values [56, 79]. The high carbon values coupled with the arid environment makes camelid consumption of non-irrigated maize unlikely. However, coastal camelids might have also fed on loma vegetation, primarily composed of C4 and CAM plants, or grama salada, a salt tolerant C4 grass. Additionally, some diets with higher δ^15^N values may have also included a high percentages of fish remains, such as Peruvian hake (*Merluccius gayi peruanus*) and Peruvian anchoveta (*Engraulis ringens*), found in camelid coprolites [76]. Sea algae, with δ^15^N values below +2‰ or +3‰ (similar to terrestrial plants), cannot explain the high δ^15^N values in these archaeological remains [40].

Camelids from the highland region display intriguing patterns in nitrogen isotope values, reflecting changes in agricultural and herding practices over time. Controlling for latitudinal effects, the nitrogen isotope values (δ^1^⁵N) for highland camelids largely fall within the expected range for the consumption of fertilized agricultural plants, indicating that either intentional fertilization techniques in agricultural practices or unintentional fertilization (repeated camelid use and defecation) in pastures may have directly influenced isotopic signatures. While the carbon isotopes remain low, the enriched δ^1^⁵N values suggest that herding strategies likely involved the use of foddering C3 plants, including tubers and quinoa, which have been identified through microbotanical analyses as part of the diet of herded camelids in the highlands [22]. The increase in δ^1^⁵N values is unlikely to be the result of consumption of nitrogen-fixing plants like legumes, which are rare in the highlands and have not been identified in camelid diets. During the Early Horizon and LIP, the broad variation in δ^1^⁵N values indicates significant diversity in husbanding practices. This variation aligns with more intensive and varied agricultural practices, including the sporadic consumption of fertilized C3 crops such as tubers and traditional C3 grass pastures, which influenced dietary changes.

Our analysis partially supports the second hypothesis that the temporal intensification of maize agriculture and fertilization practices was a pivotal factor in pastoral evolution in Peru. While there is a temporal increase in nitrogen values for both coastal and highland camelids through time, this increase is not solely due to maize agriculture. The data suggest that the enrichment in δ^15^N values is not exclusively tied to increased maize cultivation but rather to a broader use of fertilization practices that enhanced nitrogen levels in various crops. For camelids residing along the coast, the rising nitrogen values reflect changing diets directly related to significant changes in agricultural techniques for maize. In contrast, camelids from the agricultural highlands maintained a more varied diet that included foddered C3 plants. Consequently, the observed isotopic shifts in camelids point to a complex interplay of evolving agricultural strategies and environmental factors influencing camelid diets over time.

### Research question 3: Isotopic evaluation of varied camelid socioeconomic roles

Our study highlights an intriguing temporal divergence in camelid diets between coastal and maize-belt areas. Among C4 taxa, maize is the most significant component in the diet of the New World archeological agrarian populations [40]. It has been identified that significant δ^13^C deviation toward C4 plant range is a sign of intentional maize feeding of camelids, as a part of organized herding following the intensification of maize agriculture [9, 10, 15]. However, the gradual intensification of maize cultivation did not uniformly influence camelid diets across these diverse regions [9, 80–83]. Coastal camelids show the anticipated shift towards a heavily maize-influenced diet while conversely, their non-coastal counterparts exhibited a decrease in carbon isotope signature. Though unexpected, this pattern appears to follow the divergent socio-economic purposes camelids filled in these distinct ecological zones.

#### Coastal camelid diet

Contemporary research has significantly revised the earlier view that camelids were exclusively herded in the Andean highlands with extensive research providing compelling evidence for the effective maintenance of herds in coastal regions [12, 76, 77, 84]. This analysis of δ^13^C and δ^15^N values in coastal camelids reveals temporal enrichment in isotopic signatures indicative of an increased consumption of maize cultivated using natural fertilizers including camelid dung and seabird guano [56, 70, 79].

Along the coast, the limited availability of forage, combined with accessible agricultural and marine resources at lower elevations, appears to have led to a reduced emphasis on camelids as a risk mitigating subsistence strategy [10, 19, 40, 76]. Indeed, subsistence herding is rare below 500 meters above sea level due to declining forage availability and increased productivity of agricultural and marine economies [11]. On the coasts, where terrestrial crop failure may be offset by marine resource use, camelid loss concurrent with maize field failure may be less damaging than in non-coastal areas. As a result, coastal camelids appear to have been more regularly foddered with maize, reflecting their cultural rather than subsistence value [41, 73, 85]. This adaptation may suggest that in coastal regions, camelids were predominantly valued for their cultural and ceremonial significance, rather than as critical components of a subsistence economy [10, 12, 19].

Within coastal societies, camelids were deeply integrated into socio-religious structures, fulfilling roles more associated with ceremonial and political functions than with day-to-day subsistence. Our temporal analysis of δ^13^C and δ^15^N values from the Early Horizon through the Late Intermediate Period shows a significant increase, indicating an intensification in the use of maize as fodder [10, 12, 16, 19]. This trend aligns with the expansion of maize agriculture as a major economic activity. Maize foddering practices, deeply rooted in the ceremonial traditions of the Chavín cult from as early as the Early Horizon period, are evidenced by significant maize consumption by camelids in the Nepeña Valley [19]. The ceremonial importance of maize continued through the Virú and Mochica periods, highlighted by enriched δ^13^C and δ^15^N values in camelid teeth, directly linking the consumption maize to funerary rituals [10, 16, 65]. This practice persisted into the Moche and Chimú periods, where maize played a crucial role in sacrificial practices at significant ceremonial sites such as Huanchaquito-Las Llamas [20, 68]. Over time, the role of maize in foddering camelids in coastal societies likely exemplified a complex interplay between agricultural practices and cultural rituals, emphasizing both camelids’ and maize’s dual role in supporting both the physical and spiritual well-being of these communities.

#### Highland camelid diet

Our analysis of camelid dietary patterns in highland regions of the Andean maize belt revealed that the diet of highland camelids predominantly relied on C3 plants from the Early Horizon to through the Late Intermediate Period. While the carbon isotope values suggest the diet of some camelids contained higher amounts of C4 and/or aquatic plants, C3 plants remained consistently the dietary base. This feeding regime remains fairly stable from the Early Horizon through the Late Intermediate Period, as indicated by low δ^13^C values and broadly stable δ^15^N values. This aligns with previous research suggesting that despite the ecological suitability for maize cultivation, there was no substantial shift towards maize-based foddering in these regions [16, 19]. The lack of carbon enrichment suggests that camelids were not fed maize, nor even brought into maize fields after harvest. This indicates that the prized crop of maize, which was ample in the maize belt, was not used for camelids. Instead, camelid diets were actively managed through grazing on C3 plants, with little inclusion of C4 plants over time.

Research on camelid herding practices in the highlands has primarily focused on the Wari Empire during the Middle Horizon (600–1000 CE). Previous studies have identified distinct herding practices across different Wari sites, highlighting differences influenced by local ecological zones and trade networks throughout the Wari Empire. Camelids from Conchopata and Uraca, which have enriched carbon values, have been identified as being fed greater quantities of maize. In contrast, camelids from Castillo de Huarmey, Quilcapampa, and Beringa had less enriched carbon isotopes, indicating a diet predominantly of C3 plants with minimal inclusion of C4 plants [9, 14, 15, 21, 22]. Building on the work of previous scholars, our analysis suggests that the majority of camelids during the Middle Horizon maintained a diet based mainly on C3 plants. This indicates widespread and stable herding practices in the highlands that did not focus on maize foddering, instead relying on C3 crops such as tubers, as identified on the dental calculus of camelid teeth [22]. While some camelids, such as those from Conchopata [9], consumed C4 plants potentially due to specialized local practices or increased trade with lower elevation areas, highland camelids consistently had less enriched carbon isotopes than those from contemporary coastal settlements, reflecting the distinct herding and agricultural practices of these regions [14].

Residents of the highlands relied on camelids as part of their subsistence economy as evidenced by the fact that most dietary protein came from domesticated camelids and agricultural crops, with less consumption of wild resources such as freshwater fish, birds, and plants [38, 59]. In the highland areas (i.e., the maize cultivation corridor), maize is a significant economic product, yet at risk of crop failure due to climatic stochasticity [2, 35, 58]. Thus, the threat of crop failure potentially favors a shift away from maize dependence for camelid fodder in these regions. Keeping camelids foddered on non-maize vegetation would help ensure that camelid herds persist in instances where maize fields fail, providing crucial subsistence and economic buffers. Our findings align with this, indicating a consistent emphasis on a diet based on C3 plants, which enhances the resilience of camelid herds and provides essential subsistence and economic stability. These observations highlight the adaptive strategies developed in response to environmental and economic challenges, characterized by a trade-off between subsistence security and the need for mobility to access dispersed resources. Adapting to marginal and variable agroecological conditions also involves economic diversification to enhance the sustainability of local communities.

## Conclusion

This study has systematically investigated the impact of the rise of maize agriculture on camelid dietary patterns in Peru from 900 BCE to 1470 CE, providing significant insights into the evolution of ancient camelid herding practices. Building on foundational isotopic research in the Andes, our findings align with and expand upon previous studies to further highlight the marked divergence in camelid dietary patterns across coastal and highland regions. This divergence arises from socio-economic differences linked to maize agriculture. In coastal areas, camelids were closely integrated into ceremonial and political roles, with diets incorporating a higher proportion of maize. Conversely, highland camelids predominantly subsisted on C3 plants, maintaining diets reflective of long-standing subsistence practices.

This analysis represents a pivotal initial regional examination of camelid herding across Peru, providing crucial baseline data for generating future research. However, achieving a comprehensive understanding of camelid herding requires further isotopic research. Future research should integrate isotopic data with other archaeological indicators such as faunal remains, systematic excavations, and broader archaeometric analyses to build a more detailed understanding of Andean camelid pastoralism. Further, as researchers have sought to challenge the traditional view that camelids were not herded on the coast there has developed a disproportionate focus on isotopic research on coastal camelids compared to their highland counterparts. This imbalance limits our regional analysis. It is vital that we extend isotopic research across various ecoregions and time periods to enhance our understanding of how camelids were utilized in religious, socio-cultural, and subsistence contexts in both highland and coastal settings, as well as the herding practices that supported these roles.

## Supporting information

S1 File The complete dataset used for our analysis. (CSV)

S2 File R markdown of our complete analysis. (HTML)
