# Supplementary material for: A cross-regional examination of camelid herding practices in Peru from 900 BCE to 1450 CE: Insights from stable isotopes in camelid bone collagen and fiber
Source: PLoS One. 2024 Oct 14;19(10):e0306205. doi: 10.1371/journal.pone.0306205 (PMC11472908; doi:10.1371/journal.pone.0306205)
Supplement: S2 File — (HTML) [file pone.0306205.s002.html]

Supplemental\_Material2.knit


# Supplemental 2

**Sarah Noe**

*2023-02-28*

This is Supplemental 2, supplemental analysis code for the manuscript
“A Cross-Regional Examination of Camelid Herding Practices in Peru from
600 BCE to 1450 CE: Insights from Stable isotopes in Camelid bone
Collagen and Fiber” submitted to PlosOne and written by Noe, Sarah J.,
Wilson, Kurt M., and McCool, Weston C. For questions about the code,
please contact Sarah J. Noe at snoe@ucsb.edu

## Results

### Working Directory

```
setwd("/Users/72sno/Documents/02_Sarah/Camelids/R")
isotope <- read.csv("SI.2.csv", fileEncoding = "latin1")
isotope.data <- subset(isotope, material != "dentine" & portion != "textile" & (is.na(isotope$`C.N`) | (`C.N` >= 2.9 & `C.N` <= 3.6)))
bone <- subset(isotope.data, material == "bone")
fiber <- subset(isotope.data, material == "fiber")  
coast <- subset(isotope.data, ecozone == "coast") 
highland <- subset(isotope.data, ecozone == "highland") 
early <- subset(isotope.data, period.categ == "early") 
late <- subset(isotope.data, period.categ == "late") 
coast.b <- subset(isotope.data, ecozone == "coast" & material == "bone") 
coast.f <- subset(isotope.data, ecozone == "coast" & material == "fiber") 
highland.b <- subset(isotope.data, ecozone == "highland" & material == "bone") 
highland.f <- subset(isotope.data, ecozone == "highland" & material == "fiber")
```

### Library

```
library(boot)
library(lme4)
```

```
## Loading required package: Matrix
```

```
library(ggplot2)
library(ggpubr)
library(gridExtra)
library(cowplot)
```

```
## 
## Attaching package: 'cowplot'
```

```
## The following object is masked from 'package:ggpubr':
## 
##     get_legend
```

```
library(visreg)
library(viridis)
```

```
## Loading required package: viridisLite
```

```
library(dplyr)
```

```
## 
## Attaching package: 'dplyr'
```

```
## The following object is masked from 'package:gridExtra':
## 
##     combine
```

```
## The following objects are masked from 'package:stats':
## 
##     filter, lag
```

```
## The following objects are masked from 'package:base':
## 
##     intersect, setdiff, setequal, union
```

```
library(readr)
```

### Question 1.1: Carbon isotopes kruskal.test

```
#We will perform a Kruskal-Wallis test to compare the δ13C values across different periods and ecozones. Below is the code and corresponding plots for the analysis.

#Bone and Fiber integrated dataset from the coast across four cultural time periods
kruskal.test(d13C.corr ~ period, data = coast)
```

```
## 
##  Kruskal-Wallis rank sum test
## 
## data:  d13C.corr by period
## Kruskal-Wallis chi-squared = 101.85, df = 3, p-value < 2.2e-16
```

```
pairwise.wilcox.test(x = coast$d13C.corr, g = coast$period)
```

```
## 
##  Pairwise comparisons using Wilcoxon rank sum test with continuity correction 
## 
## data:  coast$d13C.corr and coast$period 
## 
##                           early horizon early intermediate period
## early intermediate period 0.019         -                        
## late intermediate period  2.0e-08       < 2e-16                  
## middle horizon            0.001         0.280                    
##                           late intermediate period
## early intermediate period -                       
## late intermediate period  -                       
## middle horizon            2.4e-09                 
## 
## P value adjustment method: holm
```

```
kruskal.test(d13C.corr ~ period, data = highland)
```

```
## 
##  Kruskal-Wallis rank sum test
## 
## data:  d13C.corr by period
## Kruskal-Wallis chi-squared = 27.316, df = 3, p-value = 5.055e-06
```

```
pairwise.wilcox.test(x = highland$d13C.corr, g = highland$period)
```

```
## 
##  Pairwise comparisons using Wilcoxon rank sum test with continuity correction 
## 
## data:  highland$d13C.corr and highland$period 
## 
##                           early horizon early intermediate period
## early intermediate period 9.7e-05       -                        
## late intermediate period  1.5e-05       0.5394                   
## middle horizon            0.0014        0.0044                   
##                           late intermediate period
## early intermediate period -                       
## late intermediate period  -                       
## middle horizon            0.6362                  
## 
## P value adjustment method: holm
```

```
#Bone and Fiber integrated dataset across four cultural time periods
kruskal.test(d13C.corr ~ ecozone, data = isotope.data)
```

```
## 
##  Kruskal-Wallis rank sum test
## 
## data:  d13C.corr by ecozone
## Kruskal-Wallis chi-squared = 152.25, df = 1, p-value < 2.2e-16
```

```
pairwise.wilcox.test(x = isotope.data$d13C.corr, g = isotope.data$ecozone)
```

```
## 
##  Pairwise comparisons using Wilcoxon rank sum test with continuity correction 
## 
## data:  isotope.data$d13C.corr and isotope.data$ecozone 
## 
##          coast 
## highland <2e-16
## 
## P value adjustment method: holm
```

```
#plot results
ggplot(isotope.data, aes(x = ecozone, y = d13C.corr, fill = period)) +
  geom_boxplot(position = position_dodge(0.9)) +
  labs(title = "Period", x = "", y = "d13C") +
  scale_fill_viridis_d(option = "viridis") +
  theme_minimal()
```

```
#We will now analyze the data for early versus late time categories.
#Bone and Fiber integrated dataset Early v Late time categories
kruskal.test(d13C.corr ~ ecozone, data = isotope.data)
```

```
## 
##  Kruskal-Wallis rank sum test
## 
## data:  d13C.corr by ecozone
## Kruskal-Wallis chi-squared = 152.25, df = 1, p-value < 2.2e-16
```

```
kruskal.test(d13C.corr ~ period.categ, data = isotope.data)
```

```
## 
##  Kruskal-Wallis rank sum test
## 
## data:  d13C.corr by period.categ
## Kruskal-Wallis chi-squared = 47.803, df = 1, p-value = 4.712e-12
```

```
pairwise.wilcox.test(x = isotope.data$d13C.corr, g = isotope.data$period.categ)
```

```
## 
##  Pairwise comparisons using Wilcoxon rank sum test with continuity correction 
## 
## data:  isotope.data$d13C.corr and isotope.data$period.categ 
## 
##      early  
## late 4.7e-12
## 
## P value adjustment method: holm
```

```
#plot results
ggplot(isotope.data, aes(x = ecozone, y = d13C.corr, fill = period.categ)) +
  geom_boxplot(position = position_dodge(0.9)) +
  labs(x = "", y = "d13C") +
  scale_fill_viridis_d(option = "viridis") +
  theme_minimal()
```

```
#We will now perform Kruskal-Wallis tests for bone and fiber isotope values from both the coast and highlands.
# Krustal Wallis test for Bone and Fiber isotope values from the coast (<50masl)
kruskal.test(d13C ~ period, data = coast.b)
```

```
## 
##  Kruskal-Wallis rank sum test
## 
## data:  d13C by period
## Kruskal-Wallis chi-squared = 87.034, df = 3, p-value < 2.2e-16
```

```
kruskal.test(d13C ~ period, data = coast.f)
```

```
## 
##  Kruskal-Wallis rank sum test
## 
## data:  d13C by period
## Kruskal-Wallis chi-squared = 17.019, df = 2, p-value = 0.0002015
```

```
pairwise.wilcox.test(x = coast.b$d13C, g = coast.b$period)
```

```
## 
##  Pairwise comparisons using Wilcoxon rank sum test with continuity correction 
## 
## data:  coast.b$d13C and coast.b$period 
## 
##                           early horizon early intermediate period
## early intermediate period 0.012         -                        
## late intermediate period  4.2e-08       5.5e-15                  
## middle horizon            0.001         0.233                    
##                           late intermediate period
## early intermediate period -                       
## late intermediate period  -                       
## middle horizon            1.9e-08                 
## 
## P value adjustment method: holm
```

```
#plot results
C1 <- ggplot(coast.b, aes(x = ecozone, y = d13C, fill = period)) +
  geom_boxplot(position = position_dodge(0.9)) +
  labs(title = "Period", x = "", y = "d13C") +
  scale_fill_viridis_d(option = "viridis") +
  theme_minimal()

C2 <- ggplot(coast.b, aes(x = ecozone, y = d13C, fill = period)) +
  geom_boxplot(position = position_dodge(0.9)) +
  labs(title = "Period", x = "", y = "d13C") +
  scale_fill_viridis_d(option = "viridis") +
  theme_minimal()

# Combine plots
plot_grid(C1, C2, ncol = 2, rel_widths = c(1, 1), align = 'h')
```

```
#Krustal Wallis test for Bone isotope values from the highlands (>50masl)
kruskal.test(d13C ~ period, data = highland.b)
```

```
## 
##  Kruskal-Wallis rank sum test
## 
## data:  d13C by period
## Kruskal-Wallis chi-squared = 19.34, df = 3, p-value = 0.0002325
```

```
kruskal.test(d13C ~ period, data = highland.f)
```

```
## 
##  Kruskal-Wallis rank sum test
## 
## data:  d13C by period
## Kruskal-Wallis chi-squared = 11.046, df = 1, p-value = 0.0008888
```

```
pairwise.wilcox.test(x = highland.b$d13C, g = highland.b$period)
```

```
## Warning in wilcox.test.default(xi, xj, paired = paired, ...): cannot compute
## exact p-value with ties
## Warning in wilcox.test.default(xi, xj, paired = paired, ...): cannot compute
## exact p-value with ties
```

```
## 
##  Pairwise comparisons using Wilcoxon rank sum test with continuity correction 
## 
## data:  highland.b$d13C and highland.b$period 
## 
##                           early horizon early intermediate period
## early intermediate period 1.0000        -                        
## late intermediate period  1.5e-05       0.0075                   
## middle horizon            0.0075        0.0690                   
##                           late intermediate period
## early intermediate period -                       
## late intermediate period  -                       
## middle horizon            1.0000                  
## 
## P value adjustment method: holm
```

```
# Plot
H1 <- ggplot(highland.b, aes(x = ecozone, y = d13C, fill = period)) +
  geom_boxplot(position = position_dodge(0.9)) +
  labs(title = "Period", x = "", y = "d13C") +
  scale_fill_viridis_d(option = "viridis") +
  theme_minimal()

H2 <- ggplot(highland.f, aes(x = ecozone, y = d13C, fill = period)) +
  geom_boxplot(position = position_dodge(0.9)) +
  labs(title = "Period", x = "", y = "d13C") +
  scale_fill_viridis_d(option = "viridis") +
  theme_minimal()

# Combine plots
plot_grid(H1, H2, ncol = 2, rel_widths = c(1, 1), align = 'h')
```

### Question 2.1: Nitrogen isotopes kruskal.test

```
#In this section, we analyze the δ15N values using the Kruskal-Wallis test and pairwise Wilcoxon tests. We will examine the nitrogen isotope values across different periods and ecozones in bone and fiber samples.

#We begin by performing the Kruskal-Wallis test for δ15N values across four cultural time periods for both the coast and highland regions. Additionally, we conduct pairwise Wilcoxon tests to identify significant differences between periods.

#Bone and Fiber integrated dataset from the coast across four cultural time periods
kruskal.test(d15N.corr ~ period, data = coast)
```

```
## 
##  Kruskal-Wallis rank sum test
## 
## data:  d15N.corr by period
## Kruskal-Wallis chi-squared = 48.489, df = 3, p-value = 1.676e-10
```

```
pairwise.wilcox.test(x = coast$d15N.corr, g = coast$period)
```

```
## 
##  Pairwise comparisons using Wilcoxon rank sum test with continuity correction 
## 
## data:  coast$d15N.corr and coast$period 
## 
##                           early horizon early intermediate period
## early intermediate period 0.0028        -                        
## late intermediate period  3.0e-07       1.8e-05                  
## middle horizon            0.0028        0.7108                   
##                           late intermediate period
## early intermediate period -                       
## late intermediate period  -                       
## middle horizon            1.7e-05                 
## 
## P value adjustment method: holm
```

```
kruskal.test(d15N.corr ~ period, data = highland)
```

```
## 
##  Kruskal-Wallis rank sum test
## 
## data:  d15N.corr by period
## Kruskal-Wallis chi-squared = 11.964, df = 3, p-value = 0.007507
```

```
pairwise.wilcox.test(x = highland$d15N.corr, g = highland$period)
```

```
## 
##  Pairwise comparisons using Wilcoxon rank sum test with continuity correction 
## 
## data:  highland$d15N.corr and highland$period 
## 
##                           early horizon early intermediate period
## early intermediate period 0.135         -                        
## late intermediate period  1.000         0.022                    
## middle horizon            1.000         0.057                    
##                           late intermediate period
## early intermediate period -                       
## late intermediate period  -                       
## middle horizon            1.000                   
## 
## P value adjustment method: holm
```

```
#Next, we perform the Kruskal-Wallis test to compare δ15N values across different ecozones and periods in the integrated dataset. We also conduct pairwise Wilcoxon tests for these comparisons.

#Bone and Fiber integrated dataset across four cultural time periods
kruskal.test(d15N.corr ~ ecozone, data = isotope.data)
```

```
## 
##  Kruskal-Wallis rank sum test
## 
## data:  d15N.corr by ecozone
## Kruskal-Wallis chi-squared = 22.144, df = 1, p-value = 2.529e-06
```

```
pairwise.wilcox.test(x = isotope.data$d15N.corr, g = isotope.data$ecozone)
```

```
## 
##  Pairwise comparisons using Wilcoxon rank sum test with continuity correction 
## 
## data:  isotope.data$d15N.corr and isotope.data$ecozone 
## 
##          coast  
## highland 2.5e-06
## 
## P value adjustment method: holm
```

```
#plot results
ggplot(isotope.data, aes(x = ecozone, y = d15N.corr, fill = period)) +
  geom_boxplot(position = position_dodge(0.9)) +
  labs(title = "Period", x = "", y = "d15N") +
  scale_fill_viridis_d(option = "viridis") +
  theme_minimal()
```

```
#We then analyze the δ15N values by comparing early and late time categories across different ecozones.
#Bone and Fiber integrated dataset Early v Late time categories
kruskal.test(d15N.corr ~ ecozone, data = isotope.data)
```

```
## 
##  Kruskal-Wallis rank sum test
## 
## data:  d15N.corr by ecozone
## Kruskal-Wallis chi-squared = 22.144, df = 1, p-value = 2.529e-06
```

```
kruskal.test(d15N.corr ~ period.categ, data = isotope.data)
```

```
## 
##  Kruskal-Wallis rank sum test
## 
## data:  d15N.corr by period.categ
## Kruskal-Wallis chi-squared = 4.3463, df = 1, p-value = 0.03709
```

```
pairwise.wilcox.test(x = isotope.data$d15N.corr, g = isotope.data$period.categ)
```

```
## 
##  Pairwise comparisons using Wilcoxon rank sum test with continuity correction 
## 
## data:  isotope.data$d15N.corr and isotope.data$period.categ 
## 
##      early
## late 0.037
## 
## P value adjustment method: holm
```

```
#plot results
ggplot(isotope.data, aes(x = ecozone, y = d15N.corr, fill = period.categ)) +
  geom_boxplot(position = position_dodge(0.9)) +
  labs(title = "Period", x = "", y = "d15N") +
  scale_fill_viridis_d(option = "viridis") +
  theme_minimal()
```

```
#Finally, we perform Kruskal-Wallis tests for bone and fiber isotope values from both the coast and highlands.
# Krustal Wallis test for Bone and Fiber isotope values from the coast (<50masl)
kruskal.test(d15N ~ period, data = coast.b)
```

```
## 
##  Kruskal-Wallis rank sum test
## 
## data:  d15N by period
## Kruskal-Wallis chi-squared = 41.73, df = 3, p-value = 4.579e-09
```

```
kruskal.test(d15N ~ period, data = coast.f)
```

```
## 
##  Kruskal-Wallis rank sum test
## 
## data:  d15N by period
## Kruskal-Wallis chi-squared = 11.385, df = 2, p-value = 0.00337
```

```
pairwise.wilcox.test(x = coast.b$d15N, g = coast.b$period)
```

```
## 
##  Pairwise comparisons using Wilcoxon rank sum test with continuity correction 
## 
## data:  coast.b$d15N and coast.b$period 
## 
##                           early horizon early intermediate period
## early intermediate period 0.0398        -                        
## late intermediate period  9.5e-06       7.5e-07                  
## middle horizon            0.0096        0.2128                   
##                           late intermediate period
## early intermediate period -                       
## late intermediate period  -                       
## middle horizon            0.0039                  
## 
## P value adjustment method: holm
```

```
#plot results
C1 <- ggplot(coast.b, aes(x = ecozone, y = d15N, fill = period)) +
  geom_boxplot(position = position_dodge(0.9)) +
  labs(title = "Period", x = "", y = "d15N") +
  scale_fill_viridis_d(option = "viridis") +
  theme_minimal()

C2 <- ggplot(coast.b, aes(x = ecozone, y = d15N, fill = period)) +
  geom_boxplot(position = position_dodge(0.9)) +
  labs(title = "Period", x = "", y = "d15N") +
  scale_fill_viridis_d(option = "viridis") +
  theme_minimal()

# Combine plots
plot_grid(C1, C2, ncol = 2, rel_widths = c(1, 1), align = 'h')
```

```
#Krustal Wallis test for Bone isotope values from the highlands (>50masl)
kruskal.test(d15N ~ period, data = highland.b)
```

```
## 
##  Kruskal-Wallis rank sum test
## 
## data:  d15N by period
## Kruskal-Wallis chi-squared = 1.3098, df = 3, p-value = 0.7268
```

```
kruskal.test(d15N ~ period, data = highland.f)
```

```
## 
##  Kruskal-Wallis rank sum test
## 
## data:  d15N by period
## Kruskal-Wallis chi-squared = 0.34099, df = 1, p-value = 0.5593
```

```
pairwise.wilcox.test(x = highland.b$d15N, g = highland.b$period)
```

```
## Warning in wilcox.test.default(xi, xj, paired = paired, ...): cannot compute
## exact p-value with ties
## Warning in wilcox.test.default(xi, xj, paired = paired, ...): cannot compute
## exact p-value with ties
## Warning in wilcox.test.default(xi, xj, paired = paired, ...): cannot compute
## exact p-value with ties
```

```
## 
##  Pairwise comparisons using Wilcoxon rank sum test with continuity correction 
## 
## data:  highland.b$d15N and highland.b$period 
## 
##                           early horizon early intermediate period
## early intermediate period 1             -                        
## late intermediate period  1             1                        
## middle horizon            1             1                        
##                           late intermediate period
## early intermediate period -                       
## late intermediate period  -                       
## middle horizon            1                       
## 
## P value adjustment method: holm
```

```
#Plot
H1 <- ggplot(highland.b, aes(x = ecozone, y = d15N, fill = period)) +
  geom_boxplot(position = position_dodge(0.9)) +
  labs(title = "Period", x = "", y = "d15N") +
  scale_fill_viridis_d(option = "viridis") +
  theme_minimal()

H2 <- ggplot(highland.f, aes(x = ecozone, y = d15N, fill = period)) +
  geom_boxplot(position = position_dodge(0.9)) +
  labs(title = "Period", x = "", y = "d15N") +
  scale_fill_viridis_d(option = "viridis") +
  theme_minimal()

# Combine plots
plot_grid(H1, H2, ncol = 2, rel_widths = c(1, 1), align = 'h')
```

### Question 1.2: Carbon isotopes GLM

```
# In this section, we analyze the δ13C values using Generalized Linear Models (GLM). We will examine the effects of different factors such as altitude (masl) and cultural periods on the carbon isotope values in bone and fiber samples.
# First, we define the factor levels for the periods to ensure consistency in the analysis.

# Bone and Fiber integrated dataset
# Define factor levels for periods 
isotope.data$period <- factor(isotope.data$period, levels = c("early horizon", "early intermediate period", "middle horizon", "late intermediate period"))

# We begin by fitting three GLM models: a null model with no predictors, an additive model with altitude (masl) as the predictor, and an interaction model with both altitude and period as predictors.

# Generalized Linear Models with summary and visual regression plotting
GLM_null <- glm(d13C.corr ~ 1, data = isotope.data, family = gaussian)
summary(GLM_null)
```

```
## 
## Call:
## glm(formula = d13C.corr ~ 1, family = gaussian, data = isotope.data)
## 
## Coefficients:
##             Estimate Std. Error t value Pr(>|t|)    
## (Intercept) -15.2582     0.1265  -120.6   <2e-16 ***
## ---
## Signif. codes:  0 '***' 0.001 '**' 0.01 '*' 0.05 '.' 0.1 ' ' 1
## 
## (Dispersion parameter for gaussian family taken to be 9.446847)
## 
##     Null deviance: 5564.2  on 589  degrees of freedom
## Residual deviance: 5564.2  on 589  degrees of freedom
## AIC: 3002.3
## 
## Number of Fisher Scoring iterations: 2
```

```
GLM_additive <- glm(d13C.corr ~ masl, data = isotope.data, family = gaussian)
summary(GLM_additive)
```

```
## 
## Call:
## glm(formula = d13C.corr ~ masl, family = gaussian, data = isotope.data)
## 
## Coefficients:
##               Estimate Std. Error  t value Pr(>|t|)    
## (Intercept) -1.483e+01  1.385e-01 -107.087  < 2e-16 ***
## masl        -1.157e-03  1.772e-04   -6.528 1.44e-10 ***
## ---
## Signif. codes:  0 '***' 0.001 '**' 0.01 '*' 0.05 '.' 0.1 ' ' 1
## 
## (Dispersion parameter for gaussian family taken to be 8.823384)
## 
##     Null deviance: 5564.2  on 589  degrees of freedom
## Residual deviance: 5188.1  on 588  degrees of freedom
## AIC: 2963
## 
## Number of Fisher Scoring iterations: 2
```

```
GLM_interaction <- glm(d13C.corr ~ masl * period, data = isotope.data, family = gaussian)
summary(GLM_interaction)
```

```
## 
## Call:
## glm(formula = d13C.corr ~ masl * period, family = gaussian, data = isotope.data)
## 
## Coefficients:
##                                        Estimate Std. Error t value Pr(>|t|)    
## (Intercept)                          -1.707e+01  5.431e-01 -31.426  < 2e-16 ***
## masl                                  8.624e-04  4.251e-04   2.029   0.0430 *  
## periodearly intermediate period       1.370e+00  5.754e-01   2.380   0.0176 *  
## periodmiddle horizon                  1.078e+00  6.122e-01   1.761   0.0788 .  
## periodlate intermediate period        4.444e+00  5.797e-01   7.667 7.42e-14 ***
## masl:periodearly intermediate period -2.879e-03  5.907e-04  -4.873 1.42e-06 ***
## masl:periodmiddle horizon            -1.130e-03  4.911e-04  -2.300   0.0218 *  
## masl:periodlate intermediate period  -3.267e-03  5.314e-04  -6.147 1.46e-09 ***
## ---
## Signif. codes:  0 '***' 0.001 '**' 0.01 '*' 0.05 '.' 0.1 ' ' 1
## 
## (Dispersion parameter for gaussian family taken to be 6.563936)
## 
##     Null deviance: 5564.2  on 589  degrees of freedom
## Residual deviance: 3820.2  on 582  degrees of freedom
## AIC: 2794.4
## 
## Number of Fisher Scoring iterations: 2
```

```
# To visualize the interaction model, we use the visreg package to create a plot of δ13C values against altitude, grouped by period.
# Visual regression plotting for interaction model
visreg_plot <- visreg(GLM_interaction, "masl", by="period",
                      line = list(col = c("black")),
                      xlab = "masl", ylab = "δ13C Values",
                      cex.axis = 1.1, cex.lab = 1.2,
                      fill = list(col = "skyblue"), gg=TRUE)
visreg_plot + 
  theme(plot.background = element_rect(fill = "transparent"),
        panel.background = element_rect(fill = "transparent"),
        legend.background = element_rect(fill = "transparent"),
        panel.border = element_rect(colour = "black", fill=NA, size=1)) +
  facet_wrap(~ period, nrow = 2)
```

```
## Warning: The `size` argument of `element_rect()` is deprecated as of ggplot2 3.4.0.
## ℹ Please use the `linewidth` argument instead.
## This warning is displayed once every 8 hours.
## Call `lifecycle::last_lifecycle_warnings()` to see where this warning was
## generated.
```

```
#We compare the models using AIC and BIC to evaluate their performance.
# Model comparison metrics
aic_null <- AIC(GLM_null)
aic_additive <- AIC(GLM_additive)
aic_interaction <- AIC(GLM_interaction)

bic_null <- BIC(GLM_null)
bic_additive <- BIC(GLM_additive)
bic_interaction <- BIC(GLM_interaction)

# Output model comparison results
cat("AIC (Null Model):", aic_null, "\n")
```

```
## AIC (Null Model): 3002.298
```

```
cat("AIC (Additive Model):", aic_additive, "\n")
```

```
## AIC (Additive Model): 2963.013
```

```
cat("AIC (Interaction Model):", aic_interaction, "\n")
```

```
## AIC (Interaction Model): 2794.431
```

```
cat("BIC (Null Model):", bic_null, "\n")
```

```
## BIC (Null Model): 3011.059
```

```
cat("BIC (Additive Model):", bic_additive, "\n")
```

```
## BIC (Additive Model): 2976.154
```

```
cat("BIC (Interaction Model):", bic_interaction, "\n")
```

```
## BIC (Interaction Model): 2833.852
```

```
#Next, we analyze the bone and fiber datasets separately. We fit similar GLM models and visualize the results.
# Define factor levels once at the beginning for consistency
bone$period <- factor(bone$period, levels = c("early horizon", "early intermediate period", "middle horizon", "late intermediate period"))
fiber$period <- factor(fiber$period, levels = c("early intermediate period", "middle horizon", "late intermediate period"))

# Bone Dataset Analysis
bone$period <- as.factor(bone$period)
null_GLM_bone_carbon <- glm(d13C ~ 1, data = bone, family = gaussian)
GLM_1_bone_carbon <- glm(d13C ~ masl, data = bone, family = gaussian)
GLM_2_bone_carbon <- glm(d13C ~ masl * period, data = bone, family = gaussian)

# Summary and visual regression for bone dataset
par(mfrow = c(2, 2))
summary(GLM_2_bone_carbon)
```

```
## 
## Call:
## glm(formula = d13C ~ masl * period, family = gaussian, data = bone)
## 
## Coefficients:
##                                        Estimate Std. Error t value Pr(>|t|)    
## (Intercept)                          -1.707e+01  5.178e-01 -32.960  < 2e-16 ***
## masl                                  8.624e-04  4.053e-04   2.128   0.0339 *  
## periodearly intermediate period       1.820e+00  5.581e-01   3.261   0.0012 ** 
## periodmiddle horizon                  1.439e+00  5.966e-01   2.412   0.0163 *  
## periodlate intermediate period        4.465e+00  5.670e-01   7.874 2.86e-14 ***
## masl:periodearly intermediate period -1.419e-03  5.925e-04  -2.395   0.0171 *  
## masl:periodmiddle horizon            -1.195e-03  4.699e-04  -2.544   0.0113 *  
## masl:periodlate intermediate period  -3.276e-03  5.100e-04  -6.424 3.54e-10 ***
## ---
## Signif. codes:  0 '***' 0.001 '**' 0.01 '*' 0.05 '.' 0.1 ' ' 1
## 
## (Dispersion parameter for gaussian family taken to be 5.966973)
## 
##     Null deviance: 3523.1  on 434  degrees of freedom
## Residual deviance: 2547.9  on 427  degrees of freedom
## AIC: 2021.4
## 
## Number of Fisher Scoring iterations: 2
```

```
visreg(GLM_2_bone_carbon, "masl", by = "period", line = list(col = c("black")), 
       xlab = "masl", ylab = "δ13C Values", cex.axis = 1.1, cex.lab = 1.2, fill = list(col = "lightskyblue3"))
```

```
par(mfrow = c(1, 1))

# Fiber Dataset Analysis
fiber$period <- as.factor(fiber$period)
null_GLM_fiber_carbon <- glm(d13C ~ 1, data = fiber, family = gaussian)
GLM_1_fiber_carbon <- glm(d13C ~ masl, data = fiber, family = gaussian)
GLM_2_fiber_carbon <- glm(d13C ~ masl * period, data = fiber, family = gaussian)

# Summary and visual regression for fiber dataset
par(mfrow = c(2, 2))
summary(GLM_2_fiber_carbon)
```

```
## 
## Call:
## glm(formula = d13C ~ masl * period, family = gaussian, data = fiber)
## 
## Coefficients: (1 not defined because of singularities)
##                                       Estimate Std. Error t value Pr(>|t|)    
## (Intercept)                         -1.665e+01  4.041e-01 -41.192  < 2e-16 ***
## masl                                -7.057e-03  9.463e-04  -7.457 6.58e-12 ***
## periodmiddle horizon                -5.469e-01  1.117e+00  -0.490    0.625    
## periodlate intermediate period       2.769e+00  5.043e-01   5.491 1.67e-07 ***
## masl:periodmiddle horizon            3.202e-03  2.340e-03   1.368    0.173    
## masl:periodlate intermediate period         NA         NA      NA       NA    
## ---
## Signif. codes:  0 '***' 0.001 '**' 0.01 '*' 0.05 '.' 0.1 ' ' 1
## 
## (Dispersion parameter for gaussian family taken to be 5.035248)
## 
##     Null deviance: 1883.38  on 154  degrees of freedom
## Residual deviance:  755.29  on 150  degrees of freedom
## AIC: 697.34
## 
## Number of Fisher Scoring iterations: 2
```

```
visreg(GLM_2_fiber_carbon, "masl", by = "period", line = list(col = c("black")), 
       xlab = "masl", ylab = "δ13C Values", cex.axis = 1.1, cex.lab = 1.2, fill = list(col = "lightskyblue3"))
```

```
par(mfrow = c(1, 1))

#Finally, we compare the AIC and BIC values for the different models in both the bone and fiber datasets to determine which model best explains the variation in δ13C values.

# Bone dataset
aic_null_bone_carbon <- AIC(null_GLM_bone_carbon)
aic_GLM_1_bone_carbon <- AIC(GLM_1_bone_carbon)
aic_GLM_2_bone_carbon <- AIC(GLM_2_bone_carbon)
bic_null_bone_carbon <- BIC(null_GLM_bone_carbon)
bic_GLM_1_bone_carbon <- BIC(GLM_1_bone_carbon)
bic_GLM_2_bone_carbon <- BIC(GLM_2_bone_carbon)

cat("Bone Dataset - AIC/BIC Comparison:\n")
```

```
## Bone Dataset - AIC/BIC Comparison:
```

```
cat("AIC (Null Model):", aic_null_bone_carbon, "\n")
```

```
## AIC (Null Model): 2148.382
```

```
cat("AIC (Additive Model):", aic_GLM_1_bone_carbon, "\n")
```

```
## AIC (Additive Model): 2121.572
```

```
cat("AIC (Interaction Model):", aic_GLM_2_bone_carbon, "\n")
```

```
## AIC (Interaction Model): 2021.416
```

```
cat("BIC (Null Model):", bic_null_bone_carbon, "\n")
```

```
## BIC (Null Model): 2156.533
```

```
cat("BIC (Additive Model):", bic_GLM_1_bone_carbon, "\n")
```

```
## BIC (Additive Model): 2133.798
```

```
cat("BIC (Interaction Model):", bic_GLM_2_bone_carbon, "\n")
```

```
## BIC (Interaction Model): 2058.094
```

```
# Fiber dataset
aic_null_fiber_carbon <- AIC(null_GLM_fiber_carbon)
aic_GLM_1_fiber_carbon <- AIC(GLM_1_fiber_carbon)
aic_GLM_2_fiber_carbon <- AIC(GLM_2_fiber_carbon)
bic_null_fiber_carbon <- BIC(null_GLM_fiber_carbon)
bic_GLM_1_fiber_carbon <- BIC(GLM_1_fiber_carbon)
bic_GLM_2_fiber_carbon <- BIC(GLM_2_fiber_carbon)

cat("Fiber Dataset - AIC/BIC Comparison:\n")
```

```
## Fiber Dataset - AIC/BIC Comparison:
```

```
cat("AIC (Null Model):", aic_null_fiber_carbon, "\n")
```

```
## AIC (Null Model): 830.9673
```

```
cat("AIC (Additive Model):", aic_GLM_1_fiber_carbon, "\n")
```

```
## AIC (Additive Model): 726.553
```

```
cat("AIC (Interaction Model):", aic_GLM_2_fiber_carbon, "\n")
```

```
## AIC (Interaction Model): 697.3403
```

```
cat("BIC (Null Model):", bic_null_fiber_carbon, "\n")
```

```
## BIC (Null Model): 837.0541
```

```
cat("BIC (Additive Model):", bic_GLM_1_fiber_carbon, "\n")
```

```
## BIC (Additive Model): 735.6832
```

```
cat("BIC (Interaction Model):", bic_GLM_2_fiber_carbon, "\n")
```

```
## BIC (Interaction Model): 715.6008
```

### Question 2.2: Nitrogen isotopes LMER

```
#In this section, we analyze the δ15N values using Linear Mixed Effects Models (LMER). We will examine the effects of different factors such as altitude (masl) and cultural periods on the nitrogen isotope values in bone and fiber samples.

#First, we define the factor levels for the periods to ensure consistency in the analysis.

# Define factor levels for periods
isotope.data$period <- factor(isotope.data$period, levels = c("early horizon", "early intermediate period", "middle horizon", "late intermediate period"))

#We begin by fitting three LMER models: a null model with no predictors, an additive model with altitude (masl) as the predictor, and an interaction model with both altitude and period as predictors.

# Linear Mixed Effects Model for nitrogen isotopes
LMER_null_Nitrogen <- lmer(d15N.corr ~ 1 + (1 | latitude), data = isotope.data)
summary(LMER_null_Nitrogen)
```

```
## Linear mixed model fit by REML ['lmerMod']
## Formula: d15N.corr ~ 1 + (1 | latitude)
##    Data: isotope.data
## 
## REML criterion at convergence: 2319.7
## 
## Scaled residuals: 
##     Min      1Q  Median      3Q     Max 
## -2.2479 -0.6405 -0.0850  0.5671  4.2078 
## 
## Random effects:
##  Groups   Name        Variance Std.Dev.
##  latitude (Intercept) 0.9043   0.9509  
##  Residual             2.8185   1.6788  
## Number of obs: 590, groups:  latitude, 17
## 
## Fixed effects:
##             Estimate Std. Error t value
## (Intercept)   7.5845     0.2581   29.38
```

```
LMER_additive_Nitrogen <- lmer(d15N.corr ~ masl + (1 | latitude), data = isotope.data)
summary(LMER_additive_Nitrogen)
```

```
## Linear mixed model fit by REML ['lmerMod']
## Formula: d15N.corr ~ masl + (1 | latitude)
##    Data: isotope.data
## 
## REML criterion at convergence: 2333.3
## 
## Scaled residuals: 
##     Min      1Q  Median      3Q     Max 
## -2.2549 -0.6452 -0.0902  0.5651  4.2041 
## 
## Random effects:
##  Groups   Name        Variance Std.Dev.
##  latitude (Intercept) 0.987    0.9935  
##  Residual             2.813    1.6773  
## Number of obs: 590, groups:  latitude, 17
## 
## Fixed effects:
##               Estimate Std. Error t value
## (Intercept)  7.7816092  0.3409056  22.826
## masl        -0.0002689  0.0002748  -0.979
## 
## Correlation of Fixed Effects:
##      (Intr)
## masl -0.619
```

```
LMER_interaction_Nitrogen <- lmer(d15N.corr ~ masl * period + (1 | latitude), data = isotope.data)
summary(LMER_interaction_Nitrogen)
```

```
## Linear mixed model fit by REML ['lmerMod']
## Formula: d15N.corr ~ masl * period + (1 | latitude)
##    Data: isotope.data
## 
## REML criterion at convergence: 2363
## 
## Scaled residuals: 
##     Min      1Q  Median      3Q     Max 
## -2.2842 -0.6618 -0.0574  0.5948  4.1539 
## 
## Random effects:
##  Groups   Name        Variance Std.Dev.
##  latitude (Intercept) 1.526    1.235   
##  Residual             2.770    1.664   
## Number of obs: 590, groups:  latitude, 17
## 
## Fixed effects:
##                                        Estimate Std. Error t value
## (Intercept)                           7.0060174  0.6824291  10.266
## masl                                  0.0001495  0.0005653   0.265
## periodearly intermediate period       1.2766200  0.7898642   1.616
## periodmiddle horizon                  0.7772117  0.7948203   0.978
## periodlate intermediate period        1.3777764  1.0796141   1.276
## masl:periodearly intermediate period -0.0017763  0.0007058  -2.517
## masl:periodmiddle horizon            -0.0003942  0.0006741  -0.585
## masl:periodlate intermediate period  -0.0004448  0.0007531  -0.591
## 
## Correlation of Fixed Effects:
##             (Intr) masl   prdrip prdmdh prdlip msl:prdrip msl:ph
## masl        -0.597                                              
## prdrlyintrp -0.716  0.412                                       
## prdmddlhrzn -0.715  0.435  0.944                                
## prdltintrmp -0.633  0.375  0.457  0.459                         
## msl:prdrlip  0.482 -0.577 -0.712 -0.643 -0.311                  
## msl:prdmddh  0.440 -0.781 -0.528 -0.599 -0.308  0.642           
## msl:prdltip  0.452 -0.733 -0.335 -0.371 -0.645  0.474      0.772
```

```
#To visualize the interaction model, we use the visreg package to create a plot of δ15N values against altitude, grouped by period.
# Visual regression plotting for the interaction model
visreg_plot_Nitrogen <- visreg(LMER_interaction_Nitrogen, "masl", by="period",
                               line = list(col = c("black")),
                               xlab = "masl", ylab = "δ15N Values",
                               cex.axis = 1.1, cex.lab = 1.2,
                               fill = list(col = "skyblue"), gg=TRUE)

# Modify the plot to use facet_wrap for arranging the graphs in two rows
visreg_plot_Nitrogen + 
  theme(plot.background = element_rect(fill = "transparent"),
        panel.background = element_rect(fill = "transparent"),
        legend.background = element_rect(fill = "transparent"),
        panel.border = element_rect(colour = "black", fill=NA, size=1)) +
  facet_wrap(~ period, nrow = 2)
```

```
#We compare the models using AIC and BIC to evaluate their performance.
# Model comparison metrics
aic_null_Nitrogen <- AIC(LMER_null_Nitrogen)
aic_additive_Nitrogen <- AIC(LMER_additive_Nitrogen)
aic_interaction_Nitrogen <- AIC(LMER_interaction_Nitrogen)

bic_null_Nitrogen <- BIC(LMER_null_Nitrogen)
bic_additive_Nitrogen <- BIC(LMER_additive_Nitrogen)
bic_interaction_Nitrogen <- BIC(LMER_interaction_Nitrogen)

# Output model comparison results
cat("LMER for Nitrogen Isotopes - Model Comparison Metrics:\n")
```

```
## LMER for Nitrogen Isotopes - Model Comparison Metrics:
```

```
cat("AIC (Null Model):", aic_null_Nitrogen, "\n")
```

```
## AIC (Null Model): 2325.672
```

```
cat("AIC (Additive Model):", aic_additive_Nitrogen, "\n")
```

```
## AIC (Additive Model): 2341.312
```

```
cat("AIC (Interaction Model):", aic_interaction_Nitrogen, "\n")
```

```
## AIC (Interaction Model): 2383.021
```

```
cat("BIC (Null Model):", bic_null_Nitrogen, "\n")
```

```
## BIC (Null Model): 2338.813
```

```
cat("BIC (Additive Model):", bic_additive_Nitrogen, "\n")
```

```
## BIC (Additive Model): 2358.832
```

```
cat("BIC (Interaction Model):", bic_interaction_Nitrogen, "\n")
```

```
## BIC (Interaction Model): 2426.822
```

```
#Next, we analyze the bone and fiber datasets separately. We fit similar LMER models and visualize the results.
#Bone Dataset Analysis

bone$period <- factor(bone$period, levels = c("early horizon", "early intermediate period", "middle horizon", "late intermediate period"))

# Linear Mixed Effects Model for nitrogen isotopes in bone samples
LMER_null_bone_Nitrogen <- lmer(d15N ~ 1 + (1 | latitude), data = bone)
summary(LMER_null_bone_Nitrogen)
```

```
## Linear mixed model fit by REML ['lmerMod']
## Formula: d15N ~ 1 + (1 | latitude)
##    Data: bone
## 
## REML criterion at convergence: 1674.6
## 
## Scaled residuals: 
##     Min      1Q  Median      3Q     Max 
## -2.6141 -0.6365 -0.0701  0.4972  4.5829 
## 
## Random effects:
##  Groups   Name        Variance Std.Dev.
##  latitude (Intercept) 1.509    1.228   
##  Residual             2.524    1.589   
## Number of obs: 435, groups:  latitude, 16
## 
## Fixed effects:
##             Estimate Std. Error t value
## (Intercept)   7.8328     0.3309   23.67
```

```
LMER_additive_bone_Nitrogen <- lmer(d15N ~ masl + (1 | latitude), data = bone)
summary(LMER_additive_bone_Nitrogen)
```

```
## Linear mixed model fit by REML ['lmerMod']
## Formula: d15N ~ masl + (1 | latitude)
##    Data: bone
## 
## REML criterion at convergence: 1686.1
## 
## Scaled residuals: 
##     Min      1Q  Median      3Q     Max 
## -2.6437 -0.6351 -0.0675  0.4971  4.5806 
## 
## Random effects:
##  Groups   Name        Variance Std.Dev.
##  latitude (Intercept) 1.034    1.017   
##  Residual             2.541    1.594   
## Number of obs: 435, groups:  latitude, 16
## 
## Fixed effects:
##              Estimate Std. Error t value
## (Intercept) 7.4278151  0.3567789   20.82
## masl        0.0005389  0.0002777    1.94
## 
## Correlation of Fixed Effects:
##      (Intr)
## masl -0.615
```

```
LMER_interaction_bone_Nitrogen <- lmer(d15N ~ masl * period + (1 | latitude), data = bone)
summary(LMER_interaction_bone_Nitrogen)
```

```
## Linear mixed model fit by REML ['lmerMod']
## Formula: d15N ~ masl * period + (1 | latitude)
##    Data: bone
## 
## REML criterion at convergence: 1719.3
## 
## Scaled residuals: 
##     Min      1Q  Median      3Q     Max 
## -2.6308 -0.6400 -0.0754  0.5021  4.5945 
## 
## Random effects:
##  Groups   Name        Variance Std.Dev.
##  latitude (Intercept) 1.726    1.314   
##  Residual             2.508    1.584   
## Number of obs: 435, groups:  latitude, 16
## 
## Fixed effects:
##                                        Estimate Std. Error t value
## (Intercept)                           6.8115533  0.6932989   9.825
## masl                                  0.0010334  0.0005775   1.789
## periodearly intermediate period       0.8707460  0.7988958   1.090
## periodmiddle horizon                  0.8041258  0.8201727   0.980
## periodlate intermediate period        1.2434629  1.1275961   1.103
## masl:periodearly intermediate period -0.0016239  0.0007240  -2.243
## masl:periodmiddle horizon            -0.0005905  0.0007007  -0.843
## masl:periodlate intermediate period  -0.0007158  0.0007890  -0.907
## 
## Correlation of Fixed Effects:
##             (Intr) masl   prdrip prdmdh prdlip msl:prdrip msl:ph
## masl        -0.564                                              
## prdrlyintrp -0.670  0.360                                       
## prdmddlhrzn -0.665  0.365  0.938                                
## prdltintrmp -0.614  0.346  0.414  0.412                         
## msl:prdrlip  0.471 -0.496 -0.720 -0.670 -0.291                  
## msl:prdmddh  0.377 -0.770 -0.479 -0.521 -0.267  0.541           
## msl:prdltip  0.407 -0.729 -0.275 -0.286 -0.625  0.371      0.779
```

```
# Visual regression plotting for the interaction model in bone samples
visreg_plot_bone_Nitrogen <- visreg(LMER_interaction_bone_Nitrogen, "masl", by="period",
                                    line = list(col = c("black")),
                                    xlab = "masl", ylab = "δ15N Values",
                                    cex.axis = 1.1, cex.lab = 1.2,
                                    fill = list(col = "skyblue"), gg=TRUE)
visreg_plot_bone_Nitrogen + 
  theme(plot.background = element_rect(fill = "transparent"),
        panel.background = element_rect(fill = "transparent"),
        legend.background = element_rect(fill = "transparent"),
        panel.border = element_rect(colour = "black", fill=NA, size=1)) +
  facet_wrap(~ period, nrow = 2)
```

```
# Model comparison metrics for bone dataset
aic_null_bone_Nitrogen <- AIC(LMER_null_bone_Nitrogen)
aic_additive_bone_Nitrogen <- AIC(LMER_additive_bone_Nitrogen)
aic_interaction_bone_Nitrogen <- AIC(LMER_interaction_bone_Nitrogen)

bic_null_bone_Nitrogen <- BIC(LMER_null_bone_Nitrogen)
bic_additive_bone_Nitrogen <- BIC(LMER_additive_bone_Nitrogen)
bic_interaction_bone_Nitrogen <- BIC(LMER_interaction_bone_Nitrogen)

# Output model comparison results for bone dataset
cat("LMER for Bone Nitrogen Isotopes - Model Comparison Metrics:\n")
```

```
## LMER for Bone Nitrogen Isotopes - Model Comparison Metrics:
```

```
cat("AIC (Null Model):", aic_null_bone_Nitrogen, "\n")
```

```
## AIC (Null Model): 1680.634
```

```
cat("AIC (Additive Model):", aic_additive_bone_Nitrogen, "\n")
```

```
## AIC (Additive Model): 1694.149
```

```
cat("AIC (Interaction Model):", aic_interaction_bone_Nitrogen, "\n")
```

```
## AIC (Interaction Model): 1739.276
```

```
cat("BIC (Null Model):", bic_null_bone_Nitrogen, "\n")
```

```
## BIC (Null Model): 1692.861
```

```
cat("BIC (Additive Model):", bic_additive_bone_Nitrogen, "\n")
```

```
## BIC (Additive Model): 1710.451
```

```
cat("BIC (Interaction Model):", bic_interaction_bone_Nitrogen, "\n")
```

```
## BIC (Interaction Model): 1780.03
```

```
# Fiber isotope values across four cultural time periods
fiber$period <- factor(fiber$period, levels = c("early horizon", "early intermediate period", "middle horizon", "late intermediate period"))

# Linear Mixed Effects Model for nitrogen isotopes in fiber samples
LMER_null_fiber_Nitrogen <- lmer(d15N ~ 1 + (1 | latitude), data = fiber)
summary(LMER_null_fiber_Nitrogen)
```

```
## Linear mixed model fit by REML ['lmerMod']
## Formula: d15N ~ 1 + (1 | latitude)
##    Data: fiber
## 
## REML criterion at convergence: 513.7
## 
## Scaled residuals: 
##      Min       1Q   Median       3Q      Max 
## -2.38990 -0.63497  0.01315  0.73407  2.12123 
## 
## Random effects:
##  Groups   Name        Variance Std.Dev.
##  latitude (Intercept) 0.1072   0.3273  
##  Residual             1.5585   1.2484  
## Number of obs: 155, groups:  latitude, 4
## 
## Fixed effects:
##             Estimate Std. Error t value
## (Intercept)    8.265      0.204   40.51
```

```
LMER_additive_fiber_Nitrogen <- lmer(d15N ~ masl + (1 | latitude), data = fiber)
summary(LMER_additive_fiber_Nitrogen)
```

```
## Linear mixed model fit by REML ['lmerMod']
## Formula: d15N ~ masl + (1 | latitude)
##    Data: fiber
## 
## REML criterion at convergence: 525.7
## 
## Scaled residuals: 
##      Min       1Q   Median       3Q      Max 
## -2.37446 -0.63154  0.02758  0.74964  2.07027 
## 
## Random effects:
##  Groups   Name        Variance Std.Dev.
##  latitude (Intercept) 0.184    0.4289  
##  Residual             1.558    1.2484  
## Number of obs: 155, groups:  latitude, 4
## 
## Fixed effects:
##               Estimate Std. Error t value
## (Intercept)  8.3427734  0.3094751   26.96
## masl        -0.0003682  0.0010230   -0.36
## 
## Correlation of Fixed Effects:
##      (Intr)
## masl -0.590
```

```
LMER_interaction_fiber_Nitrogen <- lmer(d15N ~ masl * period + (1 | latitude), data = fiber)
```

```
## fixed-effect model matrix is rank deficient so dropping 1 column / coefficient
```

```
summary(LMER_interaction_fiber_Nitrogen)
```

```
## Linear mixed model fit by REML ['lmerMod']
## Formula: d15N ~ masl * period + (1 | latitude)
##    Data: fiber
## 
## REML criterion at convergence: 524.6
## 
## Scaled residuals: 
##      Min       1Q   Median       3Q      Max 
## -2.39123 -0.52431  0.03628  0.75491  2.00227 
## 
## Random effects:
##  Groups   Name        Variance Std.Dev.
##  latitude (Intercept) 0.02937  0.1714  
##  Residual             1.47080  1.2128  
## Number of obs: 155, groups:  latitude, 4
## 
## Fixed effects:
##                                  Estimate Std. Error t value
## (Intercept)                     8.9321448  0.2619557  34.098
## masl                           -0.0015627  0.0006588  -2.372
## periodmiddle horizon           -1.9242892  0.6053252  -3.179
## periodlate intermediate period -1.0008902  0.3507869  -2.853
## masl:periodmiddle horizon       0.0039814  0.0012672   3.142
## 
## Correlation of Fixed Effects:
##             (Intr) masl   prdmdh prdlip
## masl        -0.743                     
## prdmddlhrzn -0.281  0.207              
## prdltintrmp -0.719  0.518  0.202       
## msl:prdmddh  0.249 -0.301 -0.878 -0.175
## fit warnings:
## fixed-effect model matrix is rank deficient so dropping 1 column / coefficient
```

```
# Visual regression plotting for the interaction model in fiber samples
visreg_plot_fiber_Nitrogen <- visreg(LMER_interaction_fiber_Nitrogen, "masl", by="period",
                                    line = list(col = c("black")),
                                    xlab = "masl", ylab = "δ15N Values",
                                    cex.axis = 1.1, cex.lab = 1.2,
                                    fill = list(col = "skyblue"), gg=TRUE)
visreg_plot_fiber_Nitrogen + 
  theme(plot.background = element_rect(fill = "transparent"),
        panel.background = element_rect(fill = "transparent"),
        legend.background = element_rect(fill = "transparent"),
        panel.border = element_rect(colour = "black", fill=NA, size=1)) +
  facet_wrap(~ period, nrow = 2)
```

```
# Model comparison metrics for fiber dataset
aic_null_fiber_Nitrogen <- AIC(LMER_null_fiber_Nitrogen)
aic_additive_fiber_Nitrogen <- AIC(LMER_additive_fiber_Nitrogen)
aic_interaction_fiber_Nitrogen <- AIC(LMER_interaction_fiber_Nitrogen)

bic_null_fiber_Nitrogen <- BIC(LMER_null_fiber_Nitrogen)
bic_additive_fiber_Nitrogen <- BIC(LMER_additive_fiber_Nitrogen)
bic_interaction_fiber_Nitrogen <- BIC(LMER_interaction_fiber_Nitrogen)

# Output model comparison results for fiber dataset
cat("LMER for fiber Nitrogen Isotopes - Model Comparison Metrics:\n")
```

```
## LMER for fiber Nitrogen Isotopes - Model Comparison Metrics:
```

```
cat("AIC (Null Model):", aic_null_fiber_Nitrogen, "\n")
```

```
## AIC (Null Model): 519.7121
```

```
cat("AIC (Additive Model):", aic_additive_fiber_Nitrogen, "\n")
```

```
## AIC (Additive Model): 533.7204
```

```
cat("AIC (Interaction Model):", aic_interaction_fiber_Nitrogen, "\n")
```

```
## AIC (Interaction Model): 538.6319
```

```
cat("BIC (Null Model):", bic_null_fiber_Nitrogen, "\n")
```

```
## BIC (Null Model): 528.8423
```

```
cat("BIC (Additive Model):", bic_additive_fiber_Nitrogen, "\n")
```

```
## BIC (Additive Model): 545.8941
```

```
cat("BIC (Interaction Model):", bic_interaction_fiber_Nitrogen, "\n")
```

```
## BIC (Interaction Model): 559.9359
```

### Question 1.3: Carbon isotopes Boostrapping

```
#Krustal Wallis Test for Bootstrap Samples

#In this section, we perform bootstrap sampling to assess the variability in our dataset. We set a random seed for reproducibility and read in the data from a CSV file. We then sample a fraction of the data multiple times, apply filters, and perform Kruskal-Wallis tests to analyze the carbon isotope values across different periods and ecozones.

set.seed(123)
data <- read.csv("/Users/72sno/Documents/02_Sarah/Camelids/R/SI.csv")
fraction_to_sample <- 0.5

results <- replicate(1000, {
  sampled_data <- sample_frac(data, fraction_to_sample)
  isotope_data <- subset(sampled_data, material != "dentine" & portion != "textile" & 
                           (is.na(sampled_data$`C.N`) | sampled_data$`C.N` >= 2.9 & sampled_data$`C.N` <= 3.6))
  
  list(
    coast = subset(isotope_data, ecozone == "coast"),
    highland = subset(isotope_data, ecozone == "highland")
  )
}, simplify = FALSE)

results_df <- do.call(rbind, lapply(results, function(x) {
  data.frame(
    kruskal_coast_p = if (nrow(x$coast) > 1) kruskal.test(d13C.corr ~ period, data = x$coast)$p.value else NA,
    kruskal_highland_p = if (nrow(x$highland) > 1) kruskal.test(d13C.corr ~ period, data = x$highland)$p.value else NA
  )
}))

write_csv(results_df, "combined_results.csv")


#Next, we calculate the percentage of significant results for the coast and highland regions based on the p-values obtained from the Kruskal-Wallis tests. We also visualize the distribution of these p-values using histograms.
# Analysis
significant_coast <- mean(results_df$kruskal_coast_p < 0.05, na.rm = TRUE) * 100
significant_highland <- mean(results_df$kruskal_highland_p < 0.05, na.rm = TRUE) * 100

cat(sprintf("Percentage of significant results for coast: %.2f%%\n", significant_coast))
```

```
## Percentage of significant results for coast: 100.00%
```

```
cat(sprintf("Percentage of significant results for highland: %.2f%%\n", significant_highland))
```

```
## Percentage of significant results for highland: 99.60%
```

```
# Plotting
ggplot(results_df, aes(x = kruskal_coast_p)) +
  geom_histogram(bins = 50, fill = "blue", alpha = 0.7) +
  labs(title = "Distribution of p-values for Coast", x = "P-Value", y = "Frequency") +
  geom_vline(xintercept = 0.05, color = "red", linetype = "dashed")
```

```
ggplot(results_df, aes(x = kruskal_highland_p)) +
  geom_histogram(bins = 50, fill = "green", alpha = 0.7) +
  labs(title = "Distribution of p-values for Highland", x = "P-Value", y = "Frequency") +
  geom_vline(xintercept = 0.05, color = "red", linetype = "dashed")
```

```
#Wilcoxon Test for Bootstrap Samples
#In this part, we read in individual bootstrap sample files, apply filters, and perform pairwise Wilcoxon tests to analyze the carbon isotope values across different periods. We store the minimum p-values obtained from these tests and write the results to a CSV file.
results_df <- data.frame()

for (i in 1:1000) {
  filename <- sprintf("sample%04d.csv", i)
  filepath <- file.path("/Users/72sno/Documents/02_Sarah/Camelids/R/Bootstrap", filename)
  
  if (!file.exists(filepath)) {
    next  # Skip this iteration if the file does not exist
  }
  
  isotope <- read.csv(filepath, fileEncoding = "latin1")
  
  isotope.data <- subset(isotope, material != "dentine" & portion != "textile" & 
                           (is.na(isotope$`C.N`) | (`C.N` >= 2.9 & `C.N` <= 3.6)))
  coast <- subset(isotope.data, ecozone == "coast") 
  highland <- subset(isotope.data, ecozone == "highland") 
  
  if(nlevels(factor(coast$period)) > 1 && length(unique(coast$d13C.corr)) > 1) {
    wilcox_coast <- pairwise.wilcox.test(x = coast$d13C.corr, g = coast$period, p.adjust.method = "none", exact = FALSE)
    coast_p_values <- min(wilcox_coast$p.value, na.rm = TRUE)
  } else {
    coast_p_values <- NA
  }
  
  if(nlevels(factor(highland$period)) > 1 && length(unique(highland$d13C.corr)) > 1) {
    wilcox_highland <- pairwise.wilcox.test(x = highland$d13C.corr, g = highland$period, p.adjust.method = "none", exact = FALSE)
    highland_p_values <- min(wilcox_highland$p.value, na.rm = TRUE)
  } else {
    highland_p_values <- NA
  }
  
  results_row <- data.frame(
    file = filename,
    min_wilcox_coast_p = coast_p_values,
    min_wilcox_highland_p = highland_p_values
  )
  
  results_df <- rbind(results_df, results_row)
}

# Read the results file containing the p-values
write.csv(results_df, "wilcox_results.csv", row.names = FALSE)

#Finally, we calculate and print the percentage of significant results for the coast and highland regions based on the p-values obtained from the Wilcoxon tests. We also visualize the distribution of these p-values using histograms.
# Calculate the percentage of significant results
significant_coast <- sum(results_df$min_wilcox_coast_p < 0.05, na.rm = TRUE) / nrow(results_df) * 100
significant_highland <- sum(results_df$min_wilcox_highland_p < 0.05, na.rm = TRUE) / nrow(results_df) * 100

# Print the percentage of significant results
cat("Percentage of significant results for Coast:", significant_coast, "%\n")
```

```
## Percentage of significant results for Coast: 100 %
```

```
cat("Percentage of significant results for Highland:", significant_highland, "%\n")
```

```
## Percentage of significant results for Highland: 100 %
```

```
ggplot(results_df, aes(x = min_wilcox_coast_p)) +
  geom_histogram(bins = 30, fill = "blue", alpha = 0.7) +
  labs(title = "Distribution of p-values for Coast", x = "P-Value", y = "Frequency") +
  geom_vline(xintercept = 0.05, color = "red", linetype = "dashed")
```

```
ggplot(results_df, aes(x = min_wilcox_highland_p)) +
  geom_histogram(bins = 30, fill = "green", alpha = 0.7) +
  labs(title = "Distribution of p-values for Highland", x = "P-Value", y = "Frequency") +
  geom_vline(xintercept = 0.05, color = "red", linetype = "dashed")
```

### Question 2.3: Nitrogen isotopes Bootstrapping

```
#Krustal Wallis Test for Bootstrap Samples
#In this section, we perform bootstrap sampling to assess the variability in our δ15N dataset. We set a random seed for reproducibility, read in the data from a CSV file, and then sample a fraction of the data multiple times. After applying the necessary filters, we perform Kruskal-Wallis tests to analyze the nitrogen isotope values across different periods and ecozones.

set.seed(123)
data <- read.csv("/Users/72sno/Documents/02_Sarah/Camelids/R/SI.csv")
fraction_to_sample <- 0.5

results <- replicate(1000, {
  sampled_data <- sample_frac(data, fraction_to_sample)
  isotope_data <- subset(sampled_data, material != "dentine" & portion != "textile" & 
                           (is.na(sampled_data$`C.N`) | sampled_data$`C.N` >= 2.9 & sampled_data$`C.N` <= 3.6))
  
  list(
    coast = subset(isotope_data, ecozone == "coast"),
    highland = subset(isotope_data, ecozone == "highland")
  )
}, simplify = FALSE)

results_df <- do.call(rbind, lapply(results, function(x) {
  data.frame(
    kruskal_coast_p = if (nrow(x$coast) > 1) kruskal.test(d15N.corr ~ period, data = x$coast)$p.value else NA,
    kruskal_highland_p = if (nrow(x$highland) > 1) kruskal.test(d15N.corr ~ period, data = x$highland)$p.value else NA
  )
}))

write_csv(results_df, "combined_results.csv")

#Next, we calculate the percentage of significant results for the coast and highland regions based on the p-values obtained from the Kruskal-Wallis tests. We also visualize the distribution of these p-values using histograms.
# Analysis
significant_coast <- mean(results_df$kruskal_coast_p < 0.05, na.rm = TRUE) * 100
significant_highland <- mean(results_df$kruskal_highland_p < 0.05, na.rm = TRUE) * 100

cat(sprintf("Percentage of significant results for coast: %.2f%%\n", significant_coast))
```

```
## Percentage of significant results for coast: 99.90%
```

```
cat(sprintf("Percentage of significant results for highland: %.2f%%\n", significant_highland))
```

```
## Percentage of significant results for highland: 91.50%
```

```
# Plotting
ggplot(results_df, aes(x = kruskal_coast_p)) +
  geom_histogram(bins = 50, fill = "blue", alpha = 0.7) +
  labs(title = "Distribution of p-values for Coast", x = "P-Value", y = "Frequency") +
  geom_vline(xintercept = 0.05, color = "red", linetype = "dashed")
```

```
ggplot(results_df, aes(x = kruskal_highland_p)) +
  geom_histogram(bins = 50, fill = "green", alpha = 0.7) +
  labs(title = "Distribution of p-values for Highland", x = "P-Value", y = "Frequency") +
  geom_vline(xintercept = 0.05, color = "red", linetype = "dashed")
```

```
#Wilcoxon Test for Bootstrap Samples
#In this part, we read in individual bootstrap sample files, apply filters, and perform pairwise Wilcoxon tests to analyze the nitrogen isotope values across different periods. We store the minimum p-values obtained from these tests and write the results to a CSV file

results_df <- data.frame()

for (i in 1:1000) {
  filename <- sprintf("sample%04d.csv", i)
  filepath <- file.path("/Users/72sno/Documents/02_Sarah/Camelids/R/Bootstrap", filename)
  
  if (!file.exists(filepath)) {
    next  # Skip this iteration if the file does not exist
  }
  
  isotope <- read.csv(filepath, fileEncoding = "latin1")
  
  isotope.data <- subset(isotope, material != "dentine" & portion != "textile" & 
                           (is.na(isotope$`C.N`) | (`C.N` >= 2.9 & `C.N` <= 3.6)))
  coast <- subset(isotope.data, ecozone == "coast") 
  highland <- subset(isotope.data, ecozone == "highland") 
  
  if(nlevels(factor(coast$period)) > 1 && length(unique(coast$d15N.corr)) > 1) {
    wilcox_coast <- pairwise.wilcox.test(x = coast$d15N.corr, g = coast$period, p.adjust.method = "none", exact = FALSE)
    coast_p_values <- min(wilcox_coast$p.value, na.rm = TRUE)
  } else {
    coast_p_values <- NA
  }
  
  if(nlevels(factor(highland$period)) > 1 && length(unique(highland$d15N.corr)) > 1) {
    wilcox_highland <- pairwise.wilcox.test(x = highland$d15N.corr, g = highland$period, p.adjust.method = "none", exact = FALSE)
    highland_p_values <- min(wilcox_highland$p.value, na.rm = TRUE)
  } else {
    highland_p_values <- NA
  }
  
  results_row <- data.frame(
    file = filename,
    min_wilcox_coast_p = coast_p_values,
    min_wilcox_highland_p = highland_p_values
  )
  
  results_df <- rbind(results_df, results_row)
}

# Read the results file containing the p-values
write.csv(results_df, "wilcox_results.csv", row.names = FALSE)

#Finally, we calculate and print the percentage of significant results for the coast and highland regions based on the p-values obtained from the Wilcoxon tests. We also visualize the distribution of these p-values using histograms.
# Calculate the percentage of significant results
significant_coast <- sum(results_df$min_wilcox_coast_p < 0.05, na.rm = TRUE) / nrow(results_df) * 100
significant_highland <- sum(results_df$min_wilcox_highland_p < 0.05, na.rm = TRUE) / nrow(results_df) * 100

# Print the percentage of significant results
cat("Percentage of significant results for Coast:", significant_coast, "%\n")
```

```
## Percentage of significant results for Coast: 99.9 %
```

```
cat("Percentage of significant results for Highland:", significant_highland, "%\n")
```

```
## Percentage of significant results for Highland: 99.1 %
```

```
ggplot(results_df, aes(x = min_wilcox_coast_p)) +
  geom_histogram(bins = 30, fill = "blue", alpha = 0.7) +
  labs(title = "Distribution of p-values for Coast", x = "P-Value", y = "Frequency") +
  geom_vline(xintercept = 0.05, color = "red", linetype = "dashed")
```

```
ggplot(results_df, aes(x = min_wilcox_highland_p)) +
  geom_histogram(bins = 30, fill = "green", alpha = 0.7) +
  labs(title = "Distribution of p-values for Highland", x = "P-Value", y = "Frequency") +
  geom_vline(xintercept = 0.05, color = "red", linetype = "dashed")
```

### Culture Groups v Ecozone: Carbon

```
#Kruskal-Wallis Test and Visualization for Different Periods
#In this section, we perform Kruskal-Wallis tests and visualize the δ13C values for different periods and cultural groups in the dataset. The periods analyzed include the early intermediate period, middle horizon, and late intermediate period.

#Early Intermediate Period
#First, we subset the data for the early intermediate period and perform the Kruskal-Wallis test to compare δ13C values across different cultural groups.
early <- subset(isotope.data, period == "early intermediate period")  
kruskal.test(d13C.corr ~ cultural.group, data = early)
```

```
## 
##  Kruskal-Wallis rank sum test
## 
## data:  d13C.corr by cultural.group
## Kruskal-Wallis chi-squared = 33.193, df = 2, p-value = 6.199e-08
```

```
#We then create box plots to visualize the δ13C values for different cultural groups and ecozones during the early intermediate period.
E1 <- ggplot(early, aes(x = as.factor(cultural.group), y = d13C.corr, fill = cultural.group)) +
  geom_boxplot(position = position_dodge(0.9)) +
  labs(title = "", x = "", y = "") +
  scale_fill_viridis_d(option = "viridis", name = "material/period") + 
  theme_minimal() +
  theme(legend.position = "none")

E2 <- ggplot(early, aes(x = as.factor(ecozone), y = d13C.corr, fill = ecozone)) +
  geom_boxplot(position = position_dodge(0.9)) +
  labs(title = "", x = "", y = "") +
  scale_fill_viridis_d(option = "viridis", name = "material/period") + 
  theme_minimal() +
  theme(legend.position = "none")

plot_grid(E1, E2, ncol = 2, align = 'v')
```

```
#Middle Horizon
#Next, we subset the data for the middle horizon and perform the Kruskal-Wallis test to compare δ13C values across different cultural groups.
middle <- subset(isotope.data, period == "middle horizon")  
kruskal.test(d13C.corr ~ cultural.group, data = middle)
```

```
## 
##  Kruskal-Wallis rank sum test
## 
## data:  d13C.corr by cultural.group
## Kruskal-Wallis chi-squared = 12.261, df = 2, p-value = 0.002176
```

```
#We then create box plots to visualize the δ13C values for different cultural groups and ecozones during the middle horizon.
M1 <- ggplot(middle, aes(x = as.factor(cultural.group), y = d13C.corr, fill = cultural.group)) +
  geom_boxplot(position = position_dodge(0.9)) +
  labs(title = "", x = "", y = "") +
  scale_fill_viridis_d(option = "viridis", name = "material/period") + 
  theme_minimal() +
  theme(legend.position = "none")

M2 <- ggplot(middle, aes(x = as.factor(ecozone), y = d13C.corr, fill = ecozone)) +
  geom_boxplot(position = position_dodge(0.9)) +
  labs(title = "", x = "", y = "") +
  scale_fill_viridis_d(option = "viridis", name = "material/period") + 
  theme_minimal() +
  theme(legend.position = "none")

plot_grid(M1, M2, ncol = 2, align = 'v')
```

```
#Late Intermediate Period
#Finally, we subset the data for the late intermediate period and perform the Kruskal-Wallis test to compare δ13C values across different cultural groups.

late <- subset(isotope.data, period == "late intermediate period")  
kruskal.test(d13C.corr ~ cultural.group, data = late)
```

```
## 
##  Kruskal-Wallis rank sum test
## 
## data:  d13C.corr by cultural.group
## Kruskal-Wallis chi-squared = 19.806, df = 2, p-value = 5.003e-05
```

```
#We then create box plots to visualize the δ13C values for different cultural groups and ecozones during the late intermediate period.
L1 <- ggplot(late, aes(x = as.factor(cultural.group), y = d13C.corr, fill = cultural.group)) +
  geom_boxplot(position = position_dodge(0.9)) +
  labs(title = "", x = "", y = "") +
  scale_fill_viridis_d(option = "viridis", name = "material/period") + 
  theme_minimal() +
  theme(legend.position = "none")

L2 <- ggplot(late, aes(x = as.factor(ecozone), y = d13C.corr, fill = ecozone)) +
  geom_boxplot(position = position_dodge(0.9)) +
  labs(title = "", x = "", y = "") +
  scale_fill_viridis_d(option = "viridis", name = "material/period") + 
  theme_minimal() +
  theme(legend.position = "none")

plot_grid(L1, L2, ncol = 2, align = 'v')
```

### Culture Groups v Ecozone: Nitrogen

```
#Kruskal-Wallis Test and Visualization for Different Periods
#In this section, we perform Kruskal-Wallis tests and visualize the δ15N values for different periods and cultural groups in the dataset. The periods analyzed include the early intermediate period, middle horizon, and late intermediate period.

#Early Intermediate Period
#First, we subset the data for the early intermediate period and perform the Kruskal-Wallis test to compare δ15N values across different cultural groups.
early <- subset(isotope.data, period == "early intermediate period")  

kruskal.test(d15N.corr ~ cultural.group, data = early)
```

```
## 
##  Kruskal-Wallis rank sum test
## 
## data:  d15N.corr by cultural.group
## Kruskal-Wallis chi-squared = 13.193, df = 2, p-value = 0.001365
```

```
#We then create box plots to visualize the δ15N values for different cultural groups and ecozones during the early intermediate period.
E1 <- ggplot(early, aes(x = as.factor(cultural.group), y = d15N.corr, fill = cultural.group)) +
  geom_boxplot(position = position_dodge(0.9)) +
  labs(title = "", x = "", y = "") +
  scale_fill_viridis_d(option = "viridis", name = "material/period") + 
  theme_minimal() +
  theme(legend.position = "none")

E2 <- ggplot(early, aes(x = as.factor(ecozone), y = d15N.corr, fill = ecozone)) +
  geom_boxplot(position = position_dodge(0.9)) +
  labs(title = "", x = "", y = "") +
  scale_fill_viridis_d(option = "viridis", name = "material/period") + 
  theme_minimal() +
  theme(legend.position = "none")

plot_grid(E1, E2, ncol = 2, align = 'v')
```

```
#Middle Horizon
#Next, we subset the data for the middle horizon and perform the Kruskal-Wallis test to compare δ15N values across different cultural groups.
middle <- subset(isotope.data, period == "middle horizon")  
kruskal.test(d15N.corr ~ cultural.group, data = middle)
```

```
## 
##  Kruskal-Wallis rank sum test
## 
## data:  d15N.corr by cultural.group
## Kruskal-Wallis chi-squared = 0.71464, df = 2, p-value = 0.6995
```

```
#We then create box plots to visualize the δ15N values for different cultural groups and ecozones during the middle horizon.
M1 <- ggplot(middle, aes(x = as.factor(cultural.group), y = d15N.corr, fill = cultural.group)) +
  geom_boxplot(position = position_dodge(0.9)) +
  labs(title = "", x = "", y = "") +
  scale_fill_viridis_d(option = "viridis", name = "material/period") + 
  theme_minimal() +
  theme(legend.position = "none")

M2 <- ggplot(middle, aes(x = as.factor(ecozone), y = d15N.corr, fill = ecozone)) +
  geom_boxplot(position = position_dodge(0.9)) +
  labs(title = "", x = "", y = "") +
  scale_fill_viridis_d(option = "viridis", name = "material/period") + 
  theme_minimal() +
  theme(legend.position = "none")

plot_grid(M1, M2, ncol = 2, align = 'v')
```

```
#Late Intermediate Period
#Finally, we subset the data for the late intermediate period and perform the Kruskal-Wallis test to compare δ15N values across different cultural groups.
late <- subset(isotope.data, period == "late intermediate period" & cultural.group != "Recuay")  
kruskal.test(d15N.corr ~ cultural.group, data = late)
```

```
## 
##  Kruskal-Wallis rank sum test
## 
## data:  d15N.corr by cultural.group
## Kruskal-Wallis chi-squared = 0.96991, df = 2, p-value = 0.6157
```

```
#We then create box plots to visualize the δ15N values for different cultural groups and ecozones during the late intermediate period.
L1 <- ggplot(late, aes(x = as.factor(cultural.group), y = d15N.corr, fill = cultural.group)) +
  geom_boxplot(position = position_dodge(0.9)) +
  labs(title = "", x = "", y = "") +
  scale_fill_viridis_d(option = "viridis", name = "material/period") + 
  theme_minimal() +
  theme(legend.position = "none")

L2 <- ggplot(late, aes(x = as.factor(ecozone), y = d15N.corr, fill = ecozone)) +
  geom_boxplot(position = position_dodge(0.9)) +
  labs(title = "", x = "", y = "") +
  scale_fill_viridis_d(option = "viridis", name = "material/period") + 
  theme_minimal() +
  theme(legend.position = "none")

plot_grid(L1, L2, ncol = 2, align = 'v')
```

### Latitudinal Effect

```
#Latitudinal Effect on δ15N Values
#In this section, we analyze the effect of latitude on the δ15N values using Generalized Linear Models (GLM). We first fit a GLM with latitude as a predictor and then compare it to a null model.
# Initial GLM with latitude as the predictor
GLM1 <- glm(d15N ~ latitude, data = isotope.data)
summary(GLM1)
```

```
## 
## Call:
## glm(formula = d15N ~ latitude, data = isotope.data)
## 
## Coefficients:
##             Estimate Std. Error t value Pr(>|t|)    
## (Intercept)  7.09193    0.32262  21.982   <2e-16 ***
## latitude    -0.04861    0.03022  -1.608    0.109    
## ---
## Signif. codes:  0 '***' 0.001 '**' 0.01 '*' 0.05 '.' 0.1 ' ' 1
## 
## (Dispersion parameter for gaussian family taken to be 2.773921)
## 
##     Null deviance: 767.23  on 275  degrees of freedom
## Residual deviance: 760.05  on 274  degrees of freedom
## AIC: 1068.8
## 
## Number of Fisher Scoring iterations: 2
```

```
visreg(GLM1, "latitude", scale="response", partial=FALSE, line=list(col="black"), fill=list(col="lightskyblue3"))
```

```
# Null model
null_model_GLM1 <- glm(d15N ~ 1, data = isotope.data)
summary(null_model_GLM1)
```

```
## 
## Call:
## glm(formula = d15N ~ 1, data = isotope.data)
## 
## Coefficients:
##             Estimate Std. Error t value Pr(>|t|)    
## (Intercept)   7.5851     0.1005   75.44   <2e-16 ***
## ---
## Signif. codes:  0 '***' 0.001 '**' 0.01 '*' 0.05 '.' 0.1 ' ' 1
## 
## (Dispersion parameter for gaussian family taken to be 2.789925)
## 
##     Null deviance: 767.23  on 275  degrees of freedom
## Residual deviance: 767.23  on 275  degrees of freedom
## AIC: 1069.4
## 
## Number of Fisher Scoring iterations: 2
```

```
# Fit a comprehensive model with additional environmental predictors
# Ensure there are no singularities by treating categorical variables appropriately
isotope.data$elevation.Category <- factor(isotope.data$elevation.Category)
isotope.data$ecozone <- factor(isotope.data$ecozone, levels = c("coast", "highland"))

GLM_full <- glm(d15N ~ latitude + elevation.Category + ecozone, data = isotope.data)
summary(GLM_full)
```

```
## 
## Call:
## glm(formula = d15N ~ latitude + elevation.Category + ecozone, 
##     data = isotope.data)
## 
## Coefficients: (1 not defined because of singularities)
##                            Estimate Std. Error t value Pr(>|t|)    
## (Intercept)                 6.54769    0.61438  10.657   <2e-16 ***
## latitude                   -0.11651    0.07189  -1.621    0.106    
## elevation.Category500-3500 -0.57052    0.54813  -1.041    0.299    
## ecozonehighland                  NA         NA      NA       NA    
## ---
## Signif. codes:  0 '***' 0.001 '**' 0.01 '*' 0.05 '.' 0.1 ' ' 1
## 
## (Dispersion parameter for gaussian family taken to be 2.773077)
## 
##     Null deviance: 767.23  on 275  degrees of freedom
## Residual deviance: 757.05  on 273  degrees of freedom
## AIC: 1069.7
## 
## Number of Fisher Scoring iterations: 2
```

```
# Extract residuals to correct for environmental influences
corrected_d15N <- residuals(GLM_full)
isotope.data$corrected_d15N <- corrected_d15N

# Reanalyze the relationship between latitude and corrected δ15N values
GLM_corrected <- glm(corrected_d15N ~ latitude, data = isotope.data)
summary(GLM_corrected)
```

```
## 
## Call:
## glm(formula = corrected_d15N ~ latitude, data = isotope.data)
## 
## Coefficients:
##               Estimate Std. Error t value Pr(>|t|)
## (Intercept)  1.530e-15  3.220e-01       0        1
## latitude    -1.753e-16  3.016e-02       0        1
## 
## (Dispersion parameter for gaussian family taken to be 2.762957)
## 
##     Null deviance: 757.05  on 275  degrees of freedom
## Residual deviance: 757.05  on 274  degrees of freedom
## AIC: 1067.7
## 
## Number of Fisher Scoring iterations: 2
```

```
visreg(GLM_corrected, "latitude", scale="response", partial=FALSE, line=list(col="black"), fill=list(col="lightskyblue3"))
```

### Juvenile v Adult d15N

```
setwd("/Users/72sno/Documents/02_Sarah/Camelids/R")
isotope <- read.csv("SI.2.csv", fileEncoding = "latin1")

# Define the selected sites and age categories
selected_sites <- c("El Castillo de Huarmey", "Huaca Cao Viejo", "Huaca Santa Clara", "Huanchaquito", "Upanca")
selected_age_categ <- c("adult", "juvenile")

# Subset the data to exclude unwanted materials and portions, and filter for valid C:N ratio
SI.juv <- isotope %>%
  filter(site %in% selected_sites & 
           age.categ %in% selected_age_categ)

# Kruskal-Wallis test to compare d15N values between age categories within each site
kruskal_results <- SI.juv %>%
  group_by(site) %>%
  summarise(p.value = kruskal.test(d15N.corr ~ age.categ)$p.value)

print(kruskal_results)
```

```
## # A tibble: 5 × 2
##   site                   p.value
##   <chr>                    <dbl>
## 1 El Castillo de Huarmey   0.870
## 2 Huaca Cao Viejo          0.669
## 3 Huaca Santa Clara        0.174
## 4 Huanchaquito             0.599
## 5 Upanca                   0.208
```

```
# Wilcoxon rank-sum test to compare d15N values between age categories for each site using normal approximation
wilcox_results <- SI.juv %>%
  group_by(site) %>%
  summarise(p.value = wilcox.test(d15N.corr ~ age.categ, exact = FALSE)$p.value)

print(wilcox_results)
```

```
## # A tibble: 5 × 2
##   site                   p.value
##   <chr>                    <dbl>
## 1 El Castillo de Huarmey   0.896
## 2 Huaca Cao Viejo          0.693
## 3 Huaca Santa Clara        0.181
## 4 Huanchaquito             0.603
## 5 Upanca                   0.231
```

```
# Generate the boxplot
ggplot(SI.juv, aes(x = site, y = d15N.corr, fill = age.categ)) +
  geom_boxplot(position = position_dodge(0.9)) +
  theme(axis.text.x = element_text(angle = 45, hjust = 1)) +
  labs(x = "Site", y = "d15N values", title = "Boxplot of d15N values by site")
```

### Scatterplot of Carbon v Nitrogen

```
# Extend the shape vector to include 26 values
shape_vector <- c(1:25, 0)  # Adding one more shape

# Create the ggplot
ggplot(isotope, aes(x = d13C.corr, y = d15N.corr, color = site)) +
  geom_point(aes(shape = site), size = 3.5) + 
  scale_shape_manual(values = shape_vector) + 
  theme_classic(base_size= 16) +
  labs(x="13C Values", y="15N Values") +  
  theme(legend.position = "bottom", legend.background = element_rect(fill = NULL), legend.title = element_text(size = 8), legend.text = element_text(size = 8)) + 
  scale_x_continuous(breaks = seq(-24, -5, 1), limits=c(-25, -2)) +
  scale_y_continuous(breaks = seq(3, 14, 1), limits=c(1, 16)) +
  labs(
    title = 'Scatter plot of carbon and nitrogen by site'
  )
```

```
## Warning: Removed 1 row containing missing values or values outside the scale range
## (`geom_point()`).
```
